# Topological suppression of quantum tunnelling in a lanthanide single-ion molecular magnet

**DOI:** 10.1038/s41467-026-74798-z

**Published:** 2026-06-26

**Authors:** Sagar Paul, Eufemio Moreno-Pineda, Concepción Molina-Jirón, Appu Sunil, Mario Ruben, Anupam Garg, Wolfgang Wernsdorfer

**Affiliations:** 1 Physikalisches Institut, Karlsruhe Institute of Technology, Karlsruhe, Germany; 2 Depto. de Química-Física, Universidad de Panamá, Facultad de Ciencias Naturales, Exactas y Tecnología, Panamá, Panamá; 3 Grupo de Investigación de Materiales, Universidad de Panamá, Facultad de Ciencias Naturales, Exactas y Tecnología, Panamá, Panamá; 4 Institute of Quantum Materials and Technologies (IQMT), Karlsruhe Institute of Technology (KIT), Eggenstein-Leopoldshafen, Germany; 5 Depto. de Bioquímica, Escuela de Química, Facultad de Ciencias Naturales, Exactas y Tecnología, Universidad de Panamá, Panamá, Panamá; 6 Institute of Nanotechnology (INT), Karlsruhe Institute of Technology (KIT), Eggenstein-Leopoldshafen, Germany; 7 Centre Européen de Sciences Quantiques (CESQ), Institutde Science et d’Ingénierie Supramoléculaires (ISIS), Strasbourg, Cedex France; 8 Department of Physics and Astronomy, Northwestern University, Evanston, IL USA

**Keywords:** Magnetic properties and materials, Condensed-matter physics

## Abstract

Quantum coherence can be preserved by exploiting topology, encoding information in global geometric properties that resist local perturbations. These properties depend on the trajectory of quantum operations and curvature in parameter space, offering a topology-based route to fault-tolerant quantum computation. While geometric phase interference (Berry phase) is widely studied to probe a system’s topology, its direct detection in 4f-based molecular magnets—promising qudit platforms—has remained elusive. We present a magneto-spectroscopic μSQUID-EPR approach to resolve tunnel splittings in the Gd-based molecular magnet [^160^GdPc₂]⁻ (Pc = phthalocyanine). By irradiating single crystals with microwaves under transverse magnetic fields, we map the spin (*S* = 7/2) manifold and observe pronounced oscillations in tunnel splitting—a hallmark of quantum phase interference. These oscillations reveal topological quenching and higher-order anisotropy, underscoring the role of topology in 4f systems and opening pathways toward holonomic quantum computation.

## Abstract (web summary)

Quantum phase interference in molecular magnets could provide a route to topological control of spin dynamics but has remained inaccessible in lanthanide systems. Here, the authors report its observation in a 4f single-ion molecular magnet using magneto-spectroscopy, resolving tunnelling splittings and revealing topological quenching.

## Introduction

Topological protection, a cornerstone of modern quantum physics, manifests across diverse platforms^1–5^—from the dissipationless edge states of the quantum Hall effect^6–8^ to the elusive Majorana modes in topological superconductors^9–13^. In molecular spin systems, such protection can emerge through geometric phase interference (Berry phase) between quantum tunnelling paths^14–18^. To apply this principle in holonomic quantum computation^19–24^ (HQC)—where quantum gates are implemented via topological operations—uncovering the underlying topological structure of molecular magnets (MMs) is essential. Quantum phase interference (QPI) in MMs has been observed under transverse magnetic fields^14,17,25^, serving as a probe of spin topology, revealing regimes of topologically suppressed quantum tunnelling of magnetisation (QTM) due to destructive geometric-phase interference. These regimes correspond to the diabolical points^26–30^ (DPs)—singularities in parameter space where energy surfaces meet, and upon encircling which the wavefunction acquires a Berry phase—providing critical insight into the system’s topological landscape. Beyond their relevance to fault-tolerant qubits, the existence of DPs offers a means of externally controlling QTM via transverse magnetic fields, thereby modulating the magnetisation dynamics central to the MM characteristics.

Amongst systems exhibiting exotic topological features, MMs are particularly compelling due to their rich quantum behaviour, which has led to their proposal for several technological schemes^31,32^. Lanthanide-based (4f) MMs have been proposed for high-density data storage^33–35^ and quantum information processing^36–38^, owing to their strong anisotropy and long coherence times. Yet, directly detecting Berry phases in 4f systems remains an outstanding experimental challenge. By leveraging the Landau-Zener tunnelling rates through measured µSQUID magnetisation jumps, it was possible to observe the Berry phase in 3d(transition metal)-MMs^14,39^. However, this approach fails in 4f-MMs, where the tunnel gaps exceed the millikelvin range, resulting in broad, overlapping transitions in *M*(*H*) that could not be resolved into individual splittings. We overcome this limitation by employing µSQUID-EPR^40,41^, a magneto-spectroscopic approach to directly map the spin states and resolve the tunnel splitting. A Gd-based MM, Et_4_N[^160^GdPc_2_] (where Pc = phthalocyanine, and Et_4_N = tetraethyl ammonium), with a single ^160^Gd^3+^ ion as the sole magnetic core, a well-characterised spin Hamiltonian, and a microwave-accessible spin manifold, is studied as a platform for exploring topology-driven QTM. Irradiating the microcrystal with microwave pulses while collecting the *M*(*H*) loops allows us to explore the energy manifold of the system. Ultimately, this method permitted us to capture not only the tunnel splitting but also the full response of the spin manifold to transverse magnetic fields. Observation of transverse-field-driven oscillations in tunnel splitting, spanning 5–200 mK, is thus achieved. These oscillations bear the signature of QPI or topological quenching of quantum tunnelling and highlight the significant role of transverse magnetic fields on magnetisation dynamics of 4f-MMs. We observe a spin topology only partially explored to date, particularly the sign-dependent influence of fourth-order transverse anisotropy terms on the topological structure and dynamics. Our findings emphasise the importance of exploring the inherent QTM in 4f-MMs, as well as the spin topology via the DPs or the topologically suppressed QTM. Harnessing these topological details of spin orientation space in MMs could unlock a robust, geometry-driven pathway to fault-tolerant quantum computation—surpassing even their well-recognised coherent behaviour.

## Results and discussion

### µSQUID-EPR mapping of tunnelling gaps

**Fig. 1:**
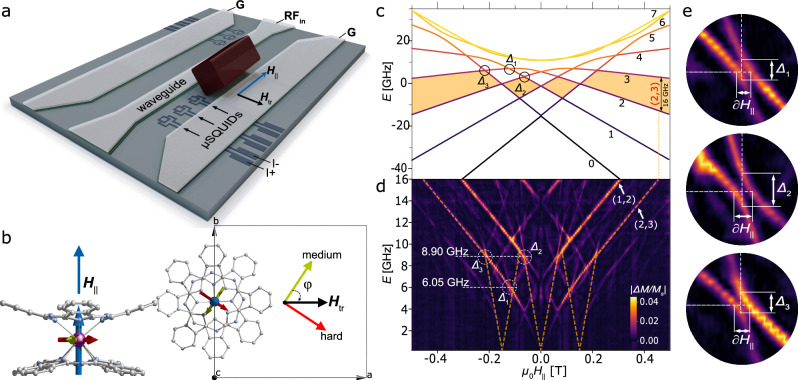
Frequency-dependent µSQUID-EPR Investigation. **a** Schematic figure of µSQUID-EPR chip comprising the µSQUID loops and the coplanar waveguide for microwave irradiation. **b** Crystallographic orientation of the [^160^GdPc_2_]^−^ complex with the easy axis (blue arrow), medium (green), and hard (red) axes, and crystal packing of the molecules, showing the orientation of the molecule in the unit cell along the *c*-crystallographic axis (right panel). Colour code: Gd, purple; N, cyan; C, grey. Hydrogens and the Et_4_N^+^ counter cation are omitted for clarity. **c** Zeeman diagram for the [^160^GdPc_2_]^−^ complex as determined via µSQUID-EPR studies and parameters as described in the text. The three marked tunnel splitting can be identified as Δ_1_: (*m*_s_ =) −5/2 → + 1/2, Δ_2_: −5/2 → + 3/2 and Δ_3_: −7/2 → + 1/2. The numbers from 0 to 7 correspond to the energy levels from the lowest to the highest in energy. **d** Frequency map (Δ*M*(*H*_**||**_,ν)) with the easy axis applied along the easy axes of the crystal. The frequencies shown in this panel correspond to the energy positions for the investigation of spin interference effects. **e** Zoomed region for each explored tunnel gap.

The multilevel character of Et_4_N[^160^GdPc_2_] (or [^160^GdPc_2_]^−^) (*S* = 7/2, *L* = 0, *I* = 0), the sizable anisotropy, and the crystal packing, make this system an excellent test bed for QPI effects. In contrast to 3d-based MMs, where QPI is observable via µSQUID studies, the exceedingly large tunnelling gaps in a 4f-MM require a different approach. We, henceforth, exploit the resonant absorption observed in the field-orientation dependent *M*(*H*) curves upon microwave absorption in the frequency range of *ν* = 0.1–20 GHz employing µSQUID-EPR technique (Fig. 1a). For this purpose, a single micro-crystal of [^160^GdPc_2_]^−^ was mounted on the µSQUID-EPR chip and measured at a base temperature of 30 mK. The crystal was aligned so that the easy axis and preferably also the hard axis (see below) of the MM are parallel to the applied fields, that is, *µ*_0_**H**_||_ along the easy axis and *µ*_0_**H**_tr_ along the hard axis or at least within the hard–medium plane (Fig. 1a). This alignment is often challenging and typically requires multiple placement attempts, as the external vector field is strictly confined to the µSQUID plane (see “Methods”). Figure 1c shows the µSQUID-EPR “frequency map” (see “Methods”) and the corresponding Zeeman diagram for [^160^GdPc_2_]^−^ with *µ*_0_**H**_||_ precisely along the easy axis and *µ*_0_**H**_tr_ = 0. As a pre-requisite for this study, the µSQUID-EPR allows precise determination of spin Hamiltonian parameters^40^.

The field-dependent frequency map (Δ*M*(*H*_**||**_,ν)) exhibits several resonant absorption peaks, all of which can be fitted employing by the following axial parameters: ${B}_{2}^{0}$ = −680.3(5) MHz, ${B}_{4}^{0}$ = −1.45(1) MHz and *g* = 2.0 (see Figs. 1c, d and S3), for a Hamiltonian of the form (1):

1 $${{{H}}}_{{{\rm{Gd}}}}=g{\mu }_{B}{\mu }_{0}{{\bf{H}}}{{\boldsymbol{.}}}\hat{{{\bf{S}}}}+{\sum }_{n=1}^{3}{B}_{2n}^{0}{O}_{2n}^{0}+\left({B}_{2}^{2}{O}_{2}^{2}+{B}_{4}^{4}{O}_{4}^{4}\right).$$

here, the first term represents the electronic Zeeman interaction, while ${O}_{k}^{q}$ and ${B}_{k}^{q}$ are the Extended Stevens operators^42,43^. The second term in the equation comprises the axial ligand field parameters, while the third term consists of the two transverse ligand field parameters. The term ${B}_{6}^{0}$ was excluded from the fitting as it tends to zero. Notably, transitions originating from excited states (e.g., (2→3) in Fig. 1d) are observed, consistent with partial thermal population of these levels. Although the bath temperature is 30 mK, the relative populations of these states indicate an effective spin temperature exceeding 500 mK under microwave irradiation, with the precise value depending on the applied microwave power, pulse width, and delay (see SI Section 2).

The *µ*SQUID-EPR setup also allows the application of the field along any direction (*θ*, with respect to the easy axis of [^160^GdPc_2_]^−^) within the µSQUID plane (Fig. 1b). The presence of transverse fields results in the observation of EPR forbidden transitions with (Δ*m*_s_ ≠ 1), which carry detailed information regarding the spin Hamiltonian of the system. The fitting of the µSQUID-EPR “angular map” (Δ*M*(*H*,*θ*), see “Methods”), likewise, provides access to the transverse ligand field parameters of [^160^GdPc_2_]^−^, i.e., ${B}_{2}^{2}$ = -273(3) and ${B}_{4}^{4}$ = 3.0(3) MHz (See Fig. S4). These values are consistent with previously determined parameters^40^. The presence of ${B}_{2}^{2}$ is a consequence of reduced symmetry from the ideal *D*_*4d*_. A similar effect has been also observed in the archetypal [Mn_12_] complex^44^. Note that although the fourth‑order parameters appear small compared to the second‑order terms, a meaningful comparison should consider the scaled quantities such as ${B}_{2}^{0}{S}^{2}$ and ${B}_{4}^{0}{S}^{2}$.

The frequency map (Δ*M*(*H*_**||**_,ν) in Fig. 1a, and insets obtained with higher frequency resolution permit the resolution of the avoided crossings, i.e., hybridisation that quantifies the resonant QTM (or tunnel splitting, Δ_**i**_) between different |*m*_*s*_〉 states. The three tunnel splittings (six, including both polarities of the longitudinal field) with the highest visibilities are indicated by the circles in Fig. 1c, d. These three gaps correspond to Δ_1_ = |−5/2〉 →|+1/2〉, Δ_2_ =  |−5/2〉 →|+3/2〉 and Δ_3_ = |−7/2〉 →|+1/2〉. We denote these as: −*m*_s_ → + *m*_s_–*n*, hence, for Δ_1_: −*m*_s_ = −5/2 → *m*_s_–*n* = ½ (i.e., *n* = 2 (even parity)), and similarly for Δ_2_: *n* = 1 (odd Parity) and Δ_3_: *n* = 3 (odd Parity), as also described in earlier works^14,39^. Here, the *m*_s_ are the diabatic state labels, the true eigenstates are mixed and do not correspond uniquely to these *m*_s_ values. Denoting the change of spin at each transition as Δ*m*_i_, we have Δ*m*_1_ = 3 at Δ_1_ and Δ*m*_2,3_ = 4 at Δ_2,3_.

**Fig. 2:**
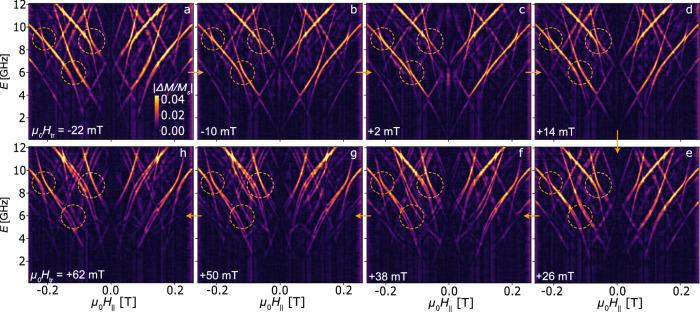
Transverse field frequency-map variation. Frequency map (Δ*M*(*H*_**||**_,ν)) variation upon transverse field (**H**_tr_) application, highlighting the oscillating behaviour of Δ_1–3_ at **H**_tr_ **a** −22 mT, **b** −10 mT, **c** +2 mT, **d** +14 mT, **e** +26 mT, **f** +38 mT, **g** +50 mT, and **h** +62 mT. The frequency maps were collected with the field (**H**_||_) applied along the easy axes of the crystal.

A major advance with respect to our previous work^40^, however, is the direct experimental observation of how the entire spin manifold (Zeeman diagram) of the MM, including Δ_i_, reacts to transverse fields. *Frequency maps* at different **H**_tr_ were collected with **H**_tr_ aligned nearly along the hard axis of the MM. This is presented as an animation (SI. V1) comprising ~ 72 h of continuous data collected with sweep rate for **H**_||_ < 20 mT/s (adiabatic sweep), 0.1 GHz steps in frequency, and 4 mT steps in **H**_tr_. Some of the frames, captured in Fig. 2 at different constant **H**_tr_, indicate the non-trivial oscillations in Δ_i_(*H*_tr_). At certain **H**_tr_ values, the gap Δ_1_ (even *n*) closes, while Δ_2_ (odd *n*) tends to approach its maxima, evidencing the direct signature of the parity effect (see below). The animation and Fig. 2 also reveal that the entire spin manifold’s reaction to small **H**_tr_ is mostly contributed by these oscillations of various tunnel gaps.

**Fig. 3:**
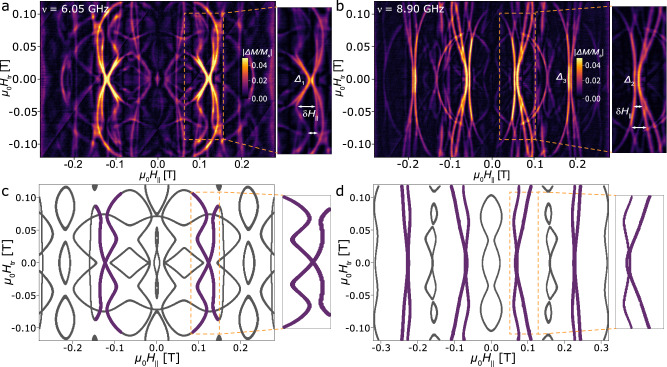
Transverse field study at fixed frequency. Transverse field dependent absorption maps (Δ*M*(*H*_||_, *H*_tr_)) between **H**_tr_ = ±120 mT at a fixed frequency of **a** 6.05 GHz and **b** 8.90 GHz. Corresponding exact numerical simulations employing Eq. (1) are shown in (**c**, **d**), respectively. The thick coloured lines and the zoomed regions highlight the oscillating behaviour of the tunnel splitting Δ_**i**_ upon **H**_tr_ variation.

Once the oscillating gaps are detected (confirming the direction of **H**_tr_), further investigation can be achieved by fixing the microwave radiation frequency (i.e., *ν* = 6.05 GHz, 8.90 GHz in Fig. 1d) and varying **H**_tr_, i.e., the absorption peak intensities (Δ*M*) plotted with **H**_||_ and **H**_tr_. Figure 3 shows the Δ*M*(*H*_||_, *H*_tr_) maps with **H**_||_ varied (at <20 mT/s) along the easy axis, in the presence of different constant **H**_tr_ values. The separations between two neighbouring absorption peaks (δ**H**_||_) associated with a Δ_i_ are approximately monotonic functions of the corresponding Δ_i_ (see Fig. 1 inset and “Methods”); hence, a study of δ**H**_||_(*H*_tr_) gives access to Δ_i_(*H*_tr_). Notably, the δ**H**_||_(*H*_tr_) clearly shows oscillatory features with the indication that the minima in Δ_1_ coincide with the maxima in Δ_2,3_ (see the enlarged sections in Fig. 3a, b).

In addition to the oscillations of *δH*_||_(*H*_tr_) corresponding to Δ_1–3_, near *H*_||_ ~ 0, the maps exhibit a pronounced tiling pattern, i.e., periodic features along the *Y* axis, due to the topological effects or QPI on the whole manifold. The oscillations of zero-longitudinal-field QTM gaps (−*m*_s_ → + *m*_s_) plausibly cause this periodic feature, as these gaps have a significant role in offsetting different states in the Zeeman diagram. These features can be quantitatively addressed by exact numerical diagonalisation of (1) under the influence of transverse fields (Fig. 3c, d). The simulations capture the periodic (tiling) features, while their enlarged sections show the oscillating gaps from the pair transitions^43,45^ (see Section 6 in SI and animated Zeeman diagrams: SI V2).

A closer inspection of Fig. 3a, b reveals that each oscillating pair of lines is accompanied by a second pair whose separation increases steadily with transverse field, indicating that the prominent features consist of one pair with an oscillating spacing *δH*_||_(*H*_tr_) and another with a monotonically increasing spacing. The same effect causes each of the resonant absorption lines in Fig. 2 to develop into two lines at higher transverse fields (see Fig. 2g), where one pair exhibits an oscillating tunnel gap and the other a monotonic increase with **H**_tr_. This behaviour is due to two molecular orientations of the unit cell, leading to a hard–medium plane alignment, with parallel easy axis arrangement (Fig. S1). At 5% dilution, it is expected that the applied **H**_tr_ aligns with the hard axis and medium axes of the statistically distributed molecules at both sites within the unit cell (see Fig. S1). However, their independent responses remain distinguishable and are advantageous in practice, as they offer additional constraints to uniquely determine the direction of **H**_tr_. For simplicity, however, we simulate and discuss one molecular orientation at a time.

### Topological quenching and parity effect

**Fig. 4:**
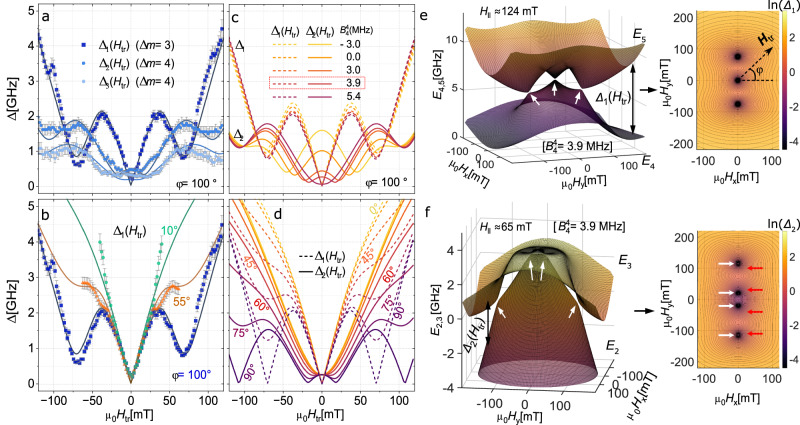
Spin parity effects and influence of transverse terms on DPs. **a** Measured tunnel splittings Δ_1,2,3_ (solid points) as a function of transverse fields, compared to the numerical simulations (lines) for *φ* = 100° (angle between **H**_tr_ and medium axis) and ${B}_{4}^{4}$ = 3.9 MHz; **b** Measured tunnel splittings Δ_1_-vs-transverse fields (solid points) for different crystal orientations, compared to the numerical simulations (lines) for ${B}_{4}^{4}$ = 3.9 MHz and *φ* = 100°, 55° and 10°, respectively; **c** Simulated tunnel splittings Δ_1,2_ as a function of transverse fields for different ${B}_{4}^{4}$ with a fixed *φ* = 100° and **d** different *φ* with a fixed ${B}_{4}^{4}$; **e**, **f** Anti-crossing energy levels simulated as a function of **H**_tr_ in the Hard-medium plane and corresponding differences (Δ_i_) as contour maps. The white, red arrows in (**f**) represent the position of the DPs with and without ${B}_{4}^{4}$ contribution. Panels a and b also show their associated error bars.

Corroboration of the topological quenching of the tunnelling gaps can be gained by rotating the crystal to align **H**_tr_ at different angles in the hard-medium plane of the MMs while maintaining the easy axis aligned with **H**_||_ (Fig. 4). When **H**_tr_ is aligned nearly along the hard axis (*φ* = 100° where *φ* denotes the angle between the medium-axis and **H**_tr_), the most prominent oscillations are observed (solid points in Fig. 4a) in Δ_1–3_(*H*_*tr*_), as extracted (see “Methods”) from the corresponding *δH*_||_(*H*_tr_) in Fig. 3a, b. On the other hand, when **H**_tr_ is not along the hard axis (*φ* = 10°), the oscillations diminish (solid points in Fig. 4b showing *Δ*_1_(*H*_tr_) for different crystal orientations and in Fig. S6 for Δ_2,3_(*H*_tr_)).

Likewise, it can be noted that the 2nd minima of Δ_1_(*H*_tr_) are nearly aligned with the maxima in Δ_2,3_(*H*_tr_), a sign of topological quenching of tunnel gaps and the parity effect. A period of ~ 70 mT is evident in Δ_1_(*H*_tr_). Only three minima are observed in Δ_1_, as only ~3 diabolic points are expected in Δ_1_ (see later). In contrast to Mn_12_ and Fe_8_ (integer *S*), the Δ_1_(*H*_tr_) with even-*n* (in −*m*_s_ → + *m*_s_–*n*) shows a minimum at zero transverse field, while Δ_2,3_(*H*_tr_) for odd-*n* exhibit maxima. This behaviour aligns with Kramer’s degeneracy for a half-integer spin system (*S* = 7/2), where even-*n* tunnel gaps must vanish at zero field.

Another difference to [Mn_12_]^39^ and [Fe_8_]^14^ is that the expected maxima in Δ_2,3_(*H*_tr_) at zero **H**_tr_ (Fig. 4a) seem to be diminished, with the minima moving towards smaller **H**_tr_. This observation is particularly compelling as topologically quenched tunnelling emerges at relatively low transverse fields, making their presence experimentally undeniable and strongly motivating a deeper investigation into the role of the 4th-order transverse ligand field parameter in spin systems.

The minima in the observed oscillations in Fig. 4 are the DPs. Encircling such a point in the transverse-field space of either of the two intersecting levels accumulates a phase of $\pi$, whereas paths that do not enclose the DP yield an accumulated phase of 0. This quantized 0/$\pi$ behavior is the Longuet–Higgins^46^ subcase of the Berry phase and serves as the topological invariant. In spin systems, several DPs (for example, 84 DPs exist for a *S* = 7/2 system^26^) for each pair of levels arise due to QPI or, in other words, topological quenching, driven by transverse magnetic fields, and transverse ligand field parameters (mainly *E* or ${B}_{2}^{2}$) that create anisotropy in the lateral plane and two dominant tunnelling paths. These paths accumulate different Berry phases, leading to interference patterns modulated by transverse fields. DPs can be modelled through methods such as the Feynman path integral (Instanton)^15,47–49^ and the discrete WKB^50–52^. The former offers some intuition on the system’s topology as it requires a classical analogue energy surface. We, therefore, employ the classical analogous energy diagram for [^160^GdPc_2_]^−^, with different signs and hypothetical magnitudes of 4th order anisotropic parameter (${B}_{4}^{4}$), by allowing the continuous orientation of the spin in Eq. (1). Figure S7 indicates that ${B}_{4}^{4}$ leaves its imprints on the instantons (classical least action paths^15,47–49^), especially its large values can lead to four instantons instead of two, as predicted for fourfold symmetry. Classical energy diagrams, however, fail to capture details of the QPI or topological quenching.

Accounting for the quantum (or semi-classical) calculations, the tunnel splittings are very much more sensitive to small ${B}_{4}^{4}$ values. The exact analytical calculations for such spin-Hamiltonians can be convoluted, as exemplified in simpler systems^52^. The predicted period of oscillations if only one axial and one transverse parameter in the Hamiltonian is considered is:

2 $$\Delta {H}_{1}=4.8\times 10^{-5}({2k}_{B}/g{{{\upmu }}}_{B})\sqrt{2{B}_{2}^{2}({B}_{2}^{2}+{3B}_{2}^{0})},$$

where ${k}_{B}$ is the Boltzmann constant, $g$ ≈ 2.00; as derived using Eq. 10 in ref. ^15^, or Eq. 2 in ref. ^14^, replacing anisotropy terms with ${B}_{2}^{0}$, ${B}_{2}^{2}$ parameters. Note that this is an incomplete theoretical description of the experimental system as the observations have indicated significant effect of 4th order parameters. For [^160^GdPc_2_]^−^ this expression yields a period ≈ 80 mT for ${B}_{2}^{0}\,=\,-680.3\,{{\rm{MHz}}},\,{B}_{2}^{2}\,=\,-273\,{{\rm{MHz}}}$, i.e., comparable to the experimentally observed periods ~ 70 mT. A ${B}_{4}^{4}$ dependent shift (toward the medium axis) of certain DPs at large transverse fields was predicted for non-zero ${B}_{2}^{0}$, ${B}_{2}^{2}$, ${B}_{4}^{4}$ (see ref. ^53^). In contrast, our observations suggest an opposite trend, i.e., the shift of certain DPs at small transverse fields. This highlights a sign-dependent role of ${B}_{4}^{4}$ not fully explored (see section 9 in SI for more details). Although several of the studies have focused^14,39^ on integer‑spin systems, the theoretical framework that describes DPs in anisotropic spin Hamiltonians is formulated for arbitrary spin values and is not intrinsically limited to integer spins. Consequently, the qualitative behaviour of DPs extends seamlessly to half‑integer systems. Except for the distinction associated with the Kramers-degeneracy—the tunnel-gaps that vanish at *H*_tr_ = 0 have even *n* for half-integer spin systems and odd *n* for the integer spin systems—the evolution of DPs follows the same symmetry principles in both classes, depending on the transverse and axial anisotropy terms. Aside from this difference, the evolution and symmetry of DPs in both classes of systems are governed by the same underlying axial and transverse anisotropy terms. Thus, our half‑integer *S* = 7/2 findings—especially the ${B}_{4}^{4}$-dependent shifts—may be compared qualitatively with the earlier works^14,39^, even though exact numerical agreement is not anticipated.

The interpretation of the experimental results of [^160^GdPc₂]^−^, hence, must consider a Hamiltonian with non-zero ${B}_{2}^{0}$, ${B}_{2}^{2}$, ${B}_{4}^{0}$, ${B}_{4}^{4}$, *H*_||_ taking into account the sign dependence of ${B}_{4}^{4}$ (relative to ${B}_{2}^{2}$). Here, we examine the DP shifts induced by the fourth‑order transverse anisotropy terms (SI, Section 9), whereas a detailed perturbative framework that includes spin‑parity and path‑dependent corrections will be provided in a subsequent theoretical analysis. In addition, to allow the inclusion of all parameters and modelling of DPs under 2D **H**_tr_, here we use the exact numerical diagonalisation. Tunnel gaps for the chosen DPs were extracted from the simulated Zeeman diagrams at different **H**_tr_ (see Section 6 in SI and SI.V2).

It is worth noting that the DPs discussed here are distinct from clock transitions^54,55^, even though both involve field-dependent extrema in energy levels and relate to decoherence resilience in different ways. Clock transitions occur when the first-order field derivative of a transition energy vanishes over a broad field-range, thereby reducing sensitivity to magnetic field-noise. By contrast, DPs are true degeneracies created by destructive quantum interference and are central to designing geometric quantum gates in molecular spin systems. Whether the clock-transition gap lies exactly between two DPs (i.e., the constructive interference point), or elsewhere, depends on its transverse-field (and transverse-parameter) dependent landscape, an aspect that merits further investigation.

### Numerical simulation of fourth-order transverse parameter effects & Berry phase

For the system studied here, numerical analysis shows that axial (${B}_{2}^{0}$) and transverse (${B}_{2}^{2}$) ligand field parameters alone cannot fully account for the observed Δ(*H*_tr_). To reach the optimum fitting, we simulate Δ_1,2_ for different hypothetical ${B}_{4}^{4}$ values (including zero) with other parameters fixed from angular and frequency maps (Fig. 4c). The transverse field angle *φ* = 100°, i.e., close to the hard axis, was chosen to match experimental data and reveals parity-dependent oscillations in Δ_1,2_. At **H**_tr_ = 0, the gap Δ_1_ (Δ*m*ᵢ = 4) is quenched while Δ_2,3_ (Δ*m*ᵢ = 3) has maxima modulated by ${B}_{4}^{4}$. Transitions with Δ*m*ᵢ = 4 are more sensitive to ${B}_{4}^{4}$ than that with Δ*m*ᵢ = 3, since the 4th order terms remain in the matrix (transition) elements involving Δ*m*_i_ = 4. Increasing ${B}_{4}^{4}$ from negative to positive values suppresses the Δ₂ maximum, indicating that a large ${B}_{4}^{4}$ with the opposite sign to ${B}_{2}^{2}$ disrupts constructive interference (at **H**_tr_ = 0) between the QTM pathways for odd *n*.

Figure 4d shows simulated Δ_1_(*H*_tr_) and Δ_2_(*H*_tr_), for various angles (*φ*) of **H**_tr_ in the hard-medium plane, using a ${B}_{4}^{4}$ value consistent with experimental data. Note that *φ* is defined relative to the medium axis; hence, *φ* = 90° shows the QPI (oscillations) most prominently, while *φ* = 0° shows more classic behaviour of Δ_1,2_(*H*_tr_). Eventually, the unique set of parameters used to fit the experimental findings in Fig. 4a and b are: *g* = 2.00 and ${B}_{2}^{0}$ = −680.3, ${B}_{4}^{0}$ = −1.45, ${B}_{2}^{2}$ = −273, ${B}_{4}^{4}$ = 3.9 MHz. Some of the pre-obtained values, acquired from angular/frequency map fitting, carried significant uncertainty. In contrast, the fittings of Δ_1,2,3_(*H*_tr_) offer excellent precision in transverse parameters, especially ${B}_{4}^{4}$, surpassing conventional orientation mapping techniques. To investigate the role of ${B}_{4}^{4}$ on topological quenching or DPs, and particularly the reason behind the contrast with a theoretical work^53^, we simulated the relevant DPs in the 2D space **H**_tr_ (hard-medium plane) and the extracted gaps between intersecting states, as shown in Fig. 4e, f. As we vary ${B}_{4}^{4}$ up to and beyond the estimated values in [^160^GdPc_2_]^−^, see Fig. S8, we find that the DPs for Δ_1_ (Δ*m*_i_ = 3) do not move with changing ${B}_{4}^{4}$, while the DPs at Δ_2_ (Δ*m*_i_ = 4) at small **H**_tr_, first move inward towards each other and then away towards the medium axis (Fig. S8m–o), in contrast to the predictions^53^. We notice that the opposite sign of ${B}_{4}^{4}$ (compared to that estimated in [^160^GdPc_2_]^−^), indeed matches the prediction in ref. ^53^ (Fig. S8p–r), i.e., the DPs at large **H**_tr_ move towards the medium axis. To clarify this sign-dependent aspect further, we simulated a tunnel gap at zero **H**_||_ for the extreme hypothetical scenarios (${B}_{4}^{4}$ > 0 and <0 with ${B}_{2}^{2}=0$) and discussed how, in the presence of non-zero ${B}_{2}^{2}$, the shift of DPs must depend on the sign of ${B}_{4}^{4}$ (see section 9 in SI). Hence, our observations point to a sign-dependent role of ${B}_{4}^{4}$, complementing the predictions in ref. ^53^, and opening the possibility of shifting DPs toward lower transverse fields **H**_tr_, thereby facilitating experimental accessibility.

Finally, to numerically confirm the topological nature of the gap minima, we evaluated the Berry phase acquired by the two energy levels near a degeneracy under a closed circuit in the transverse magnetic field. The accumulated phase was computed by numerically diagonalizing the Hamiltonian and determining the phase associated with the selected eigenstate along a circular trajectory in parameter space (see 10.5281/zenodo.19451754). When the path encircles a gap minimum, the system acquires a Berry phase of π, a result observed for several level pairs. This quantized response confirms that the gap minima correspond to true DPs displaying the Longuet–Higgins form of the Berry phase^46^.

In conclusion, we have demonstrated a magneto-spectroscopic method for the direct determination of QPI and parity effects in 4f-MMs. The technique, exploiting the resonant absorption of the *M*(*H*) loops and the ability to apply a transverse field along any direction in the x-y plane, allows not just the precise determination of the spin Hamiltonian parameters but also the controlled quenching of QTM at precisely determined transverse fields. Furthermore, the non-trivial evolution of the DPs and their dependence upon ${B}_{4}^{4}$ term can, in principle, be exploited for the design of robust MMs towards QTM, making these systems more attractive towards quantum technologies. Although the experimental techniques are insufficient to distinguish between Abelian and non-Abelian holonomies, the presence of genuine DPs suggests that more advanced parameter-space control could, in principle, enable access to non-Abelian holonomies for HQC—an aspirational yet not unreachable prospect. Hence, identifying such degeneracies in MMs is central both to simple geometric quantum gate designs and to the development of more advanced holonomic architectures in the future. Moreover, the observed motion of DPs toward experimentally accessible transverse fields suggests new synthetic strategies for chemists: by tailoring spin centre arrangements and local field environments, it may be possible to harness topologically quenched tunnelling rates. Together, these results not only deepen our understanding of spin dynamics but also chart a path forward for the rational design of next-generation quantum materials.

## Methods

### µSQUID-EPR

The µSQUID-EPR combines µSQUID magnetometry and EPR via observing resonant peaks in the magnetic signal. Microwave pulses were employed with an interval dictated by the thermal healing time of the Josephson junctions of the µSQUID (see SI). Recently, we have implemented a coplanar waveguide for µSQUID-EPR in contrast to a gold wire-based microwave antenna used before in ref. ^40^. This has significantly reduced the spin temperature while applying the microwaves, allowing precise investigation of the avoided level crossings between the spin states (see SI for further experimental details).

As shown in Fig. S2, upon excitation of microcrystals with microwave of a fixed frequency (between 0.2 and 20 GHz), resonant absorption peaks are observed in the magnetisation measured by the µSQUIDs. Figures S2 and S3 describes how the peak positions are tracked as (1) microwave frequency is varied, keeping the direction of the field fixed to the easy axis, or (2) direction of applied field is varied, keeping the microwave frequency fixed. The former yields the frequency-dependent absorption maps (“frequency maps”), while the latter yields angle-dependent absorption maps (“angular maps”). Fitting these in subsequent order provides all relevant spin Hamiltonian parameters.

### Spectroscopic route to topological quenching in MMs

To investigate the topologically quenched tunnel splittings, we probe the observable avoided crossings (Fig. 1 insets) as a function of transverse magnetic fields; however, several experimental requirements need to be ensured in a particular order.

### Step 1: alignment of the easy and hard axes

It is essential to align the longitudinal field along the easy axis, preferably with a precision better than 1°. In practice, this was done by multiple attempts of aligning the crystal’s long axis with a known on-chip (*H*_x_) direction (see Fig. 1a). Each attempt was followed by a 2D angular scan in (*H*_x_, *H*_y_) at low temperature to pinpoint the easy axis (as elaborated in the next step), along which the vector field (${H}_{\parallel }$) was finally applied (in our experiments ${H}_{\parallel }$ is not exactly along *H*_x_). Subsequent simulations validated this alignment: a few degrees of deviation at this stage leads to systematic failure of the fitting protocol, yielding unreasonable parameters or forcing the introduction of extra tilt angles as additional unknowns. The transverse field must be aligned within the hard-medium plane, while the QPI effects can be observed most prominently for transverse fields along the hard axis. The external magnetic vector fields in our experimental setup are limited within the µSQUID plane (*H*_x_, *H*_y_) since the blind mode of the µSQUID is not feasible for the discussed measurements. Hence, the single crystal needs to be placed such that both the easy and hard axes of the molecule are aligned within the µSQUID plane. The ideal cuboid shape of the [^160^GdPc_2_]^−^ single crystals, and the correlation between the molecular frame and the crystal shape axis appeared to be one of the key factors to facilitate a high probability of aligning both easy and hard axes in the measurement plane. Once the crystal is placed with appropriate orientation in the vicinity of µSQUIDs and at the trench of the coplanar waveguide (Fig. 1, schematic), the sample is cooled down to 30 mK base temperature in a dilution refrigerator for the subsequent magnetic measurements.

### Step 2: an angular map to find the easy axis

Once the microwave-induced resonant absorption peaks are observed in the magnetisation data, the next step is to obtain an *angular map* (Fig. S3) at a randomly selected frequency with high visibility of peaks. The easy axis can be precisely identified from an angular map, since the least magnitude of the resonance fields (*resfields*) indicates maximum Zeeman splittings, i.e., along the easy axis. Further, the simulation of the angular map also helps one to quickly conclude whether the hard (or medium) axis is in the 2D measurement plane or not.

### Step 3: the frequency map for easy axis and identification of avoided crossings

After a precise identification of the easy axis, the *frequency map* (Fig. 1) obtained for this fixed magnetic field direction exhibits several avoided level crossings (tunnel splittings) between −*m*_s_ → + *m*_s_–*n* states, which can be associated with that for zero transverse magnetic field. From the simulation of this map (Fig. S2), the *m*_s_ and *n* values of the tunnel splittings were identified.

### Step 4: extraction of tunnel splittings as a function of transverse fields

Next, we repeat the *frequency map* (with longitudinal field varied along the easy axis) for different constant values of transverse magnetic fields (*H*_tr_) in both polarities. A slow sweep of the longitudinal field (*H*_*||*_) (20 mT/s) was required to prominently observe the tunnel splittings and its oscillations. If the *H*_*||*_ is aligned precisely along the easy axis, the two polarities of *H*_tr_ would yield an identical effect on the tunnel splittings. The *frequency maps* at different *H*_tr_ (SI.V1) reveal whether the gaps are oscillating or just monotonically increasing, depending on the chosen direction of *H*_tr_ in the hard-medium plane. Once the direction of *H*_tr_ is confirmed, we probe the transverse field-dependent separation between two consecutive absorption peaks (δ*H*_||_) in the vicinity of an avoided crossing, and at a fixed microwave frequency that intersects the avoided crossing in the frequency map (see insets in Fig. 1). The tunnel splitting (Δ) is clearly a monotonic function of this separation δ*H*_||_, hence, its transverse field dependence is extracted from δ*H*_||_(*H*_tr_). The relation between the *δH*_||_ and Δ was approximately determined to follow the expression: Δ = $\frac{{w}_{1}{\gamma }_{1}+{{w}_{2}\gamma }_{2}}{{w}_{1}+{w}_{2}}$ *δH*_||_. Here, ${\gamma }_{{\mathrm{1,2}}}$ = d(Δ*E*_1,2_*)*/d*H* are the slopes (in high field regime of the *frequency maps*) of the two corresponding transitions meeting at the gap (see inset of Fig. S2b). The ratio of the weights ${w}_{{\mathrm{1,2}}}$ ($k$ = ${w}_{2}/{w}_{1}$) can be estimated using directly measured values from a few frequency maps using Δ = $\frac{{\gamma }_{1}+{k\gamma }_{2}}{1+k}$ *δH*_||_. In this method, the error in the estimated gap *(*${\sigma }_{\varDelta }$*)* is largely dominated by the standard deviation (${\sigma }_{k}$) in obtained $k$ as only a few maps are used, thus it is approximated as: ${\sigma }_{\varDelta }$ = $\left|{\delta H}_{{{\rm{||}}}}\,\frac{{\gamma }_{2}-{\gamma }_{1}}{{(1+k)}^{2}}\right|{\sigma }_{k}$. Using the values of Δ, *δH*_||_, and ${\gamma }_{{\mathrm{1,2}}}$ (at each tunnel-gap) from frequency maps at nine different *H*_tr_ values, we find the mean value and error in $k$ (${\sigma }_{k}$) for each tunnel-gap. We found (corresponding to the three tunnel-gaps ${\varDelta }_{{\mathrm{1,2,3}}}$) ${k}_{1}\,$= 0.41 ± 0.15, ${k}_{2}\,$= 0.77 ± 0.28 and ${k}_{3}\,$= 1.20 ± 0.61. Finally, using the above formula, we then obtain the error bars ${\sigma }_{\varDelta }$ corresponding to the data points in Fig. 4a, b.

## Supplementary information

Supplementary information

Supplementary Files

Supplementary Video 1

Supplementary Video 2

Peer Review File

## Data Availability

Supplementary information is available in the online version of the paper. The experimental data for Figs. 1–4 generated in this study have been deposited in the Zenodo database under accession code 10.5281/zenodo.20343891.

## Code availability

Custom scripts used in this work are available at: 10.5281/zenodo.19451754.
