# Supplementary material for: Topological suppression of quantum tunnelling in a lanthanide single-ion molecular magnet
Source: Nat Commun. 2026 Jun 26;17:5579. doi: 10.1038/s41467-026-74798-z (PMC13303870; doi:10.1038/s41467-026-74798-z)
Supplement: Supplementary file 1 — Supplementary information [file 41467_2026_74798_MOESM1_ESM.pdf]

Supplementary Information

Topological Suppression of Quantum Tunnelling in a Lanthanide Single-Ion Molecular Magnet

Sagar Paul[1*‡], Eufemio Moreno-Pineda[2,3,4*‡], Concepción Molina-Jirón[3,4,5], Appu Sunil[1], Mario Ruben[4,6,7], Anupam Garg[8] and Wolfgang Wernsdorfer[1,4*]

[1] *Physikalisches Institut, Karlsruhe Institute of Technology, D-76131 Karlsruhe, Germany.*

[2] *Universidad de Panamá, Facultad de Ciencias Naturales, Exactas y Tecnología, Depto. de Química-Física, 0824 Panamá, Panamá.*

[3] *Universidad de Panamá, Facultad de Ciencias Naturales, Exactas y Tecnología, Grupo de Investigación de Materiales, 0824 Panamá, Panamá.*

[4] *Institute of Quantum Materials and Technologies (IQMT), Karlsruhe Institute of Technology (KIT), Hermann-von-Helmholtz-Platz 1, D-76344, Eggenstein-Leopoldshafen, Germany.*

[5] *Depto. de Bioquímica, Escuela de Química, Facultad de Ciencias Naturales, Exactas y Tecnología, Universidad de Panamá, Panamá, Panamá.*

[6] *Institute of Nanotechnology (INT), Karlsruhe Institute of Technology (KIT), Hermann-von-Helmholtz-Platz 1, D-76344, Eggenstein-Leopoldshafen, Germany.*

[7] *Centre Européen de Sciences Quantiques (CESQ), Institutde Science et d'Ingénierie Supramoléculaires (ISIS), 8 allée Gaspard Monge, BP 70028, 67083, Strasbourg Cedex, France.*

[8] *Department of Physics and Astronomy, Northwestern University, Evanston, Illinois 60208, United States of America*

‡ *These authors contributed equally to this work.*

*Correspondence to: sagar.paul@kit.edu; eufemio.moreno@up.ac.pa; wolfgang.wernsdorfer@kit.edu

1. Electronic Structure Calculations (CASSCF)

The CASSCF-SO electronic structure calculation of $Et_4N[^{160}GdPc_2]$ was determined using *OpenMolcas*[1-3]. The CASSCF-SO calculation was performed only on the Gd^{3+} site employing the crystallographic coordinates obtained from the single crystal X-ray structure with no further optimisation. Basis sets from ANO-RCC library[4] were employed with VTZP quality for the gadolinium ion, while VDZP were employed for all remaining atoms. The molecular orbitals (MOs) were optimised in state-averaged CASSCF calculations. For this, the active space was defined by the seven 4f electrons in the seven 4f orbitals of Gd^{3+}. Four calculations were performed independently for each possible spin state, where 1 root was included for S = 7/2, 48 roots were included for S = 5/2, 392 roots were for S = 3/2 and 600 roots for S = ½ (RASSCF routine). The wavefunctions obtained from these CASSCF calculations were posteriorly mixed by spin-orbit coupling, where the state for S = 7/2 states, 48 of the S = 5/2 states, 129 of the S = 3/2 and 113 of the S = 1/2 states were included (RASSI routine)[5]. The resulting spin orbit wavefunctions were decomposed into their CF wavefunctions in the $^8S_{7/2}$ basis, employing the SINGLE_ANISO routine[6].

Table S1. Computed energy levels (the ground state is set at zero), composition of the g-tensor (g_x, g_y, g_z) and the main components (>10%) of the wavefunction for each m_J state of the ground-state multiplet $^8S_{7/2}$ for the $^{160}Gd^{3+}$ in $Et_4N[^{160}GdPc_2]$, at the CASSCF level.

Energy (cm^{-1})	g_x	g_y	g_z	Wavefunction
0	0.0000	0.0000	13.9733	100% $\|\pm7/2\rangle$
0.469	0.0551	0.0551	9.9808	100% $\|\pm5/2\rangle$
0.770	0.0155	0.0949	5.9883	100% $\|\pm3/2\rangle$
0.917	8.0244	7.9451	1.9961	100% $\|\pm1/2\rangle$

Table S2: Crystal field Hamiltonian is given as $\hat{\boldsymbol{H}}_{\boldsymbol{CF}} = \sum_{k,q} \boldsymbol{B}_{\boldsymbol{k}}^{\boldsymbol{q}} \boldsymbol{O}_{\boldsymbol{k}}^{\boldsymbol{q}}$ and the extended Stevens operator coefficients $\boldsymbol{B}_{\boldsymbol{k}}^{\boldsymbol{q}}$ are extracted from CASSCF calculations. The CF terms are highlighted in bold font.

		B_k^q (MHz)
k	*q*	**Gd^{3+}**
2	-2	0.54791
2	-1	0.00867
**2**	**0**	**-765.33279**
2	1	-0.0027
**2**	**2**	**-1.33788**
**4**	**-4**	-0.12506
4	-3	4.05544E-5
4	-2	1.04991E-4
4	-1	-4.34439E-4
**4**	**0**	**-0.2442**
4	1	1.37195E-4
4	2	0.00111
4	3	8.57266E-5
**4**	**4**	**0.32027**
6	-6	1.59333E-6
6	-5	-2.58751E-8
6	-4	2.45439E-4
6	-3	-1.42745E-6
6	-2	-8.83655E-8
6	-1	2.73502E-7
6	0	1.64931E-4
6	1	-6.20479E-7
6	2	1.94809E-7
6	3	-1.18297E-6
6	4	-3.87948E-4
6	5	-1.45462E-6
6	6	-6.30115E-7

Two differently oriented molecules of $Et_4N[^{160}GdPc_2]$ reside within the unit cell, with the second molecule being related to the first one by a -*y*, *x*, -*z* symmetry. Considering this, it is clear that the "hard" and "medium" axes are nearly 90º apart from each other (See **Figure S1**).

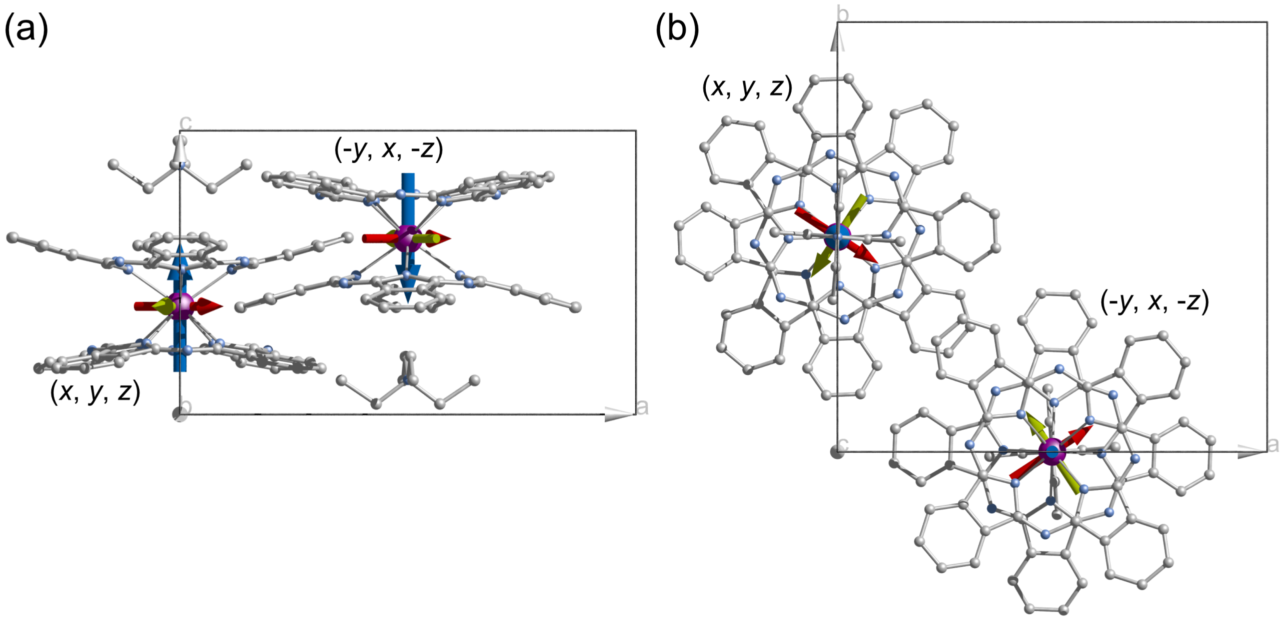

Figure S1. Packing view of the two differently oriented $Et_4N[^{160}GdPc_2]$ molecules within the unit cell and the easy (green), medium (yellow) and hard (red) axes as determined from CASSCF calculations. The molecules are view along the *b*- crystallographic axis (a) and along the *c*-crystallographic axis. Colour code: Gd, purple; N, cyan, C, grey. Hydrogens and the Et_4N^+ counter cation are omitted for clarity.

2. Experimental details for μSQUID-EPR

To facilitate microwave-induced excitation of the molecular crystal while simultaneously measuring its magnetic response, a silver coplanar waveguide was integrated atop the μSQUIDs (**Figure 1a** in main article). The μSQUIDs were patterned from a 7 nm Nb film (capped with 3 nm Si) on a sapphire substrate using e-beam lithography, which involved defining an Al mask followed by reactive-ion etching of the Nb. The overlying microwave waveguide was then fabricated by optical lithography. To achieve galvanic isolation between the Nb and the metallic waveguide, a 25 nm AlOx layer was deposited using resist undercuts and two angled depositions. This ensured that the AlOx formed a wider base (see the green spacer layer in Fig. 1a) that fully isolates the waveguide from the Nb below. A 5 nm Ti adhesion layer and a 100 nm Ag waveguide were subsequently deposited in a final orthogonal (non-tilted) evaporation step. This on-chip waveguide was wire-bonded to the coplanar waveguide of the sample holder, consisting of SMP and mini-SMP connectors for microwave input and output. To prevent excessive heating from impedance mismatch, the microwave line was not terminated at the sample holder. Instead, it was routed through the output SMP connector and externally terminated using a 50 Ω terminator block outside the dilution refrigerator. Microwave signals were generated by an AnaPico APSYN140-X frequency synthesizer, offering a broad frequency range of 100 kHz to 40 GHz with pulse modulation and an output power range spanning from -10 dBm to 25 dBm. The later could be reduced further (if needed) by using external attenuators.

As the Nb film thickness is far below the London penetration depth, flux-expulsion and vortex-trapping effects are strongly reduced, enabling operation over a wider magnetic-field range. Although the Nb and Ag layers are galvanically isolated, a capacitive coupling to the waveguide is expected, which necessitates separating microwave pulses from μSQUID readout. In practice, however, longer delays are dictated by large Josephson-healing times (see later). In the microwave-off state, the presence of the isolated Ag waveguide does not noticeably alter μSQUID behaviour (minor difference in the critical-current magnitude and its distribution).

As in standard μSQUID operation, a bias current (I_b) is applied through the current leads (I^+, I^- in **Figure 1a**) and swept to detect the critical current (I_c). The sweep begins with a sudden increase of I_b, followed by a stabilization delay (~20 μs), and then a gradual ramp until the switching event is detected. Once a sharp rise in differential resistance dV/dI is observed, the sweep is stopped and I_c is recorded. While the capacitance to the waveguide could, in principle, slow the initial current step, the long stabilization delay eliminates any practical impact. After recording I_c, a longer waiting time is required regardless (see below). As illustrated in the schematic **Figure S2a**, the microwaves were pulsed to ensure that the 'on time' did not coincide with a μSQUID measurement. This precaution was necessary as microwaves could drive the Josephson junctions (or weak links) of the μSQUID into their normal (resistive) state, disrupting the magnetization measurement. Given the minimum time required for the μSQUID to recover after each critical current detection, i.e., the Josephson healing time τ_h ~100 μs, the period was set to 300 μs, with the pulse width (ON time) minimized to ~10 μs. However, the relaxation time of molecular magnet crystals is typically much longer than these time scales. Thus, from the perspective of crystal relaxation dynamics, the microwave excitation effectively behaves as a continuous wave.

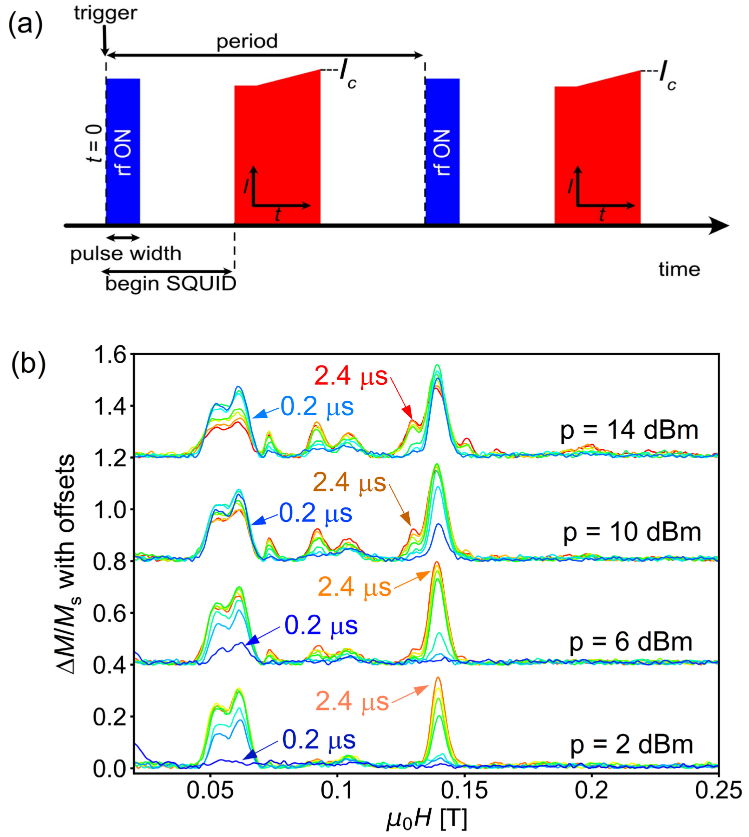

Figure S2. (a) Schematic of microwave pulse position with respect to μSQUID measurements to avoid microwave assisted heating of Josephson junctions (weak links) of the μSQUIDs. (b) Microwave absorption peak intensities as $|\Delta M/M_s|(H)$ (after baseline subtraction) are plotted for various microwave pulse width and power, while pulse period was kept fixed to 300 μS.

While the microwave pulse period (delay = 'On time' + 'Off time') is constrained to values > 150 μs due to the Josephson healing time, the optimum pulse width ('On time') and power need to be found for every single crystal. This is because the coupling of microwaves to a single crystal strongly depends on the crystal size and exact position in the trench of coplanar waveguide. The homogeneity of microwave induced field to the entire crystal also contributes to the effective coupling. **Figure S2(b)** represents how an optimum power and width is found for an individual crystal based on visibility of all peaks. The microwave absorption intensities are plotted as $|\Delta M/M_s|(H)$ (after baseline subtraction) for different values of power and width of the microwave pulse keeping the delay = 300 μs. For better visibility, the curves for different applied powers are plotted with offsets, while the curves for different pulse widths with same power are plotted together. Interestingly, the highest powers and widths do not necessarily provide highest visibility of the peaks accounted to undesired heating of the crystal. It is also found that beyond certain powers (p), the low widths (w) sometimes yield some very sharp and high peaks (see for example the curve with p = 10 dBm, w = 0.2 μs), while a neighbouring peak may not be visible. Such power and width combinations are useful to observe the transitions with optimum sharpness and minimized non-resonant absorption or heating of the crystal. However, in this study the visibility of all peaks is concerned, such that several anti-crossings are measurable in a *frequency map*. Hence, a different combination can be found best, for example, here p = 10 dBm, w = 1.6 μs yields among the highest visibilities considering all observable peaks.

3. Estimation of axial parameters from the *frequency map*

Upon precise identification of the easy axis in the measurement plane using transverse field method and an *angular map,* the field direction ($H_{||}$) is aligned with it. At 30 mK bath temperature, the $M(H_{||})$ curves in presence of microwaves at a few fixed frequencies are shown in **Figure S3a**. The peaks

identified with the arrows are found to move as a function of frequency, indicating resonant absorption of microwaves as detailed in ref 7. Here, to clarify the correlation between these $M(H_{||})$ curves with **Figure 1d** of the main article, the largest peaks for the curve at 12 GHz (blue curve) are projected on the *frequency map,* see yellow dashed lines in **Figure S3b.**

The inset in **Figure S3b** indicates the slopes of linear regimes of the anti-crossing transitions ($\gamma_{1,2}$) to explain the correlation of $\delta H_{||}$ and tunnel splitting Δ described in the 'methods' section. Note the slopes are calculated using a large range of the linear regime not entirely shown in the insets.

Figure S3c shows the Zeeman diagram (for $H_{||}$, i.e. along the easy axis) simulated using *Easyspin* (version 6.0.6)[8,9] with appropriate axial parameters obtained via fitting of multiple transitions in *the frequency map* shown in **Figure S3c**. As the transition (2,3) can be tracked with a vertical bar placed on the shaded region in **Figure S3c**, all other mentioned transitions can be tracked in the same way. The approach shown in ref. 4 employing three linear equations and the slopes at high field linear region correspond to the (7/2 → 5/2), (5/2 → 3/2) and (3/2 → 1/2) transitions leads to a unique solution of axial parameters as: g = 2.00 and B_2^0 = -685 MHz, B_4^0 = -1.45 MHz, B_6^0 = 0.004 MHz. Starting with these values, further fitting of the data (see additional MATLAB scripts (see 10.5281/zenodo.19451754)) yields the final values and errors presented in the main article.

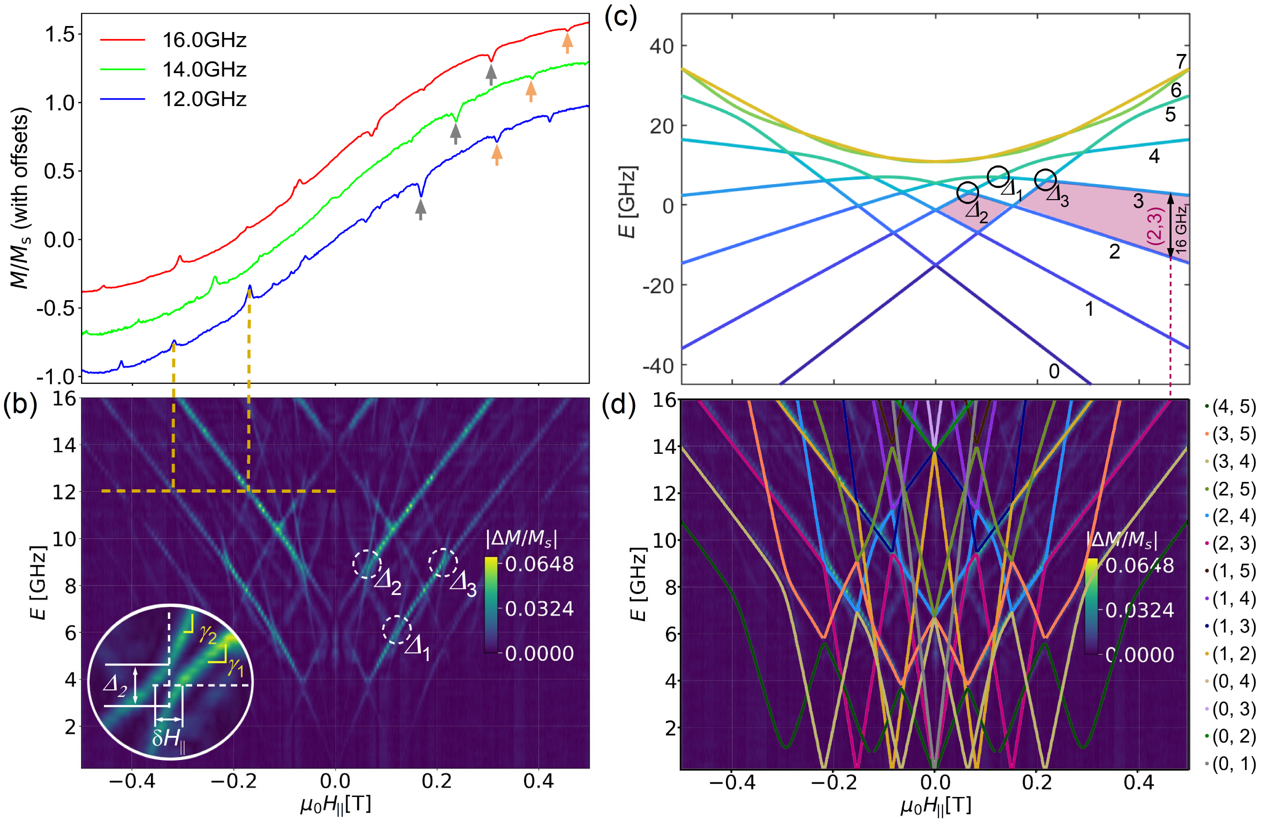

Figure S3. (a) $M(H_{||})$ curves measured at 30 mK bath temperature (with $H_{||}$ along the easy axis) in presence of microwave with fixed frequencies. (b) Microwave absorption peak intensities as $|\Delta M/M_s|(H_{||})$ (after baseline subtraction) plotted as a function of microwave frequency. Yellow dashed lines indicate the correspondence between (a) and (b). (c) The Zeeman diagram simulated for $H_{||}$ along the easy axis. The required axial parameters are obtained from the fittings of several transitions in the frequency map as shown in (d). As an example, the transition (2,3) in (d) can be tracked from the shaded region in (c).

4. Estimation of transverse parameters from an *angular map*

An *angular map* is obtained by keeping microwave frequency fixed and collecting $M(H)$ for different in-plane angles of applied field. The field-direction dependent Zeeman diagrams were simulated using *Easyspin* to theoretically obtain direction dependent resonance fields ('resfields') for the EPR transitions at a given frequency. As the axial parameters are already obtained from the *frequency*

map, an *angular map* at a randomly chosen microwave frequency (here at 8.90 GHz, see **Figure S4a**) is fitted keeping axial parameters fixed and varying only two relevant transverse ligand field parameters: B_2^2 and B_4^4. The best fitting (see additional supplementary MATLAB script (see 10.5281/zenodo.19451754)) considering the mentioned transitions (see **Figure S4b**) yields: B_2^2 = -273 ± 3 MHz and B_4^4 = 3.0 ± 0.3. Note that this fitting includes the simulations for both molecular orientations in the unit cell (**Figure S1**), as shown by individually simulating the two orientations in Figure S4(b) and (c): the first orientation has the hard axis of the system nearly aligned with H_y, while the 2nd has the medium axis nearly aligned with H_y. Clearly the angle-dependent data includes signal from both molecular orientations, and accounting to their non-interacting behaviour in diluted systems, they remain largely distinguishable. Using these parameters, together with the axial parameters found before, the fitting quality of angular maps at different microwave frequency values were also checked and found to match very well. These values are a pre-requisite to analyse further the detailed observations related to topological quenching described in this work.

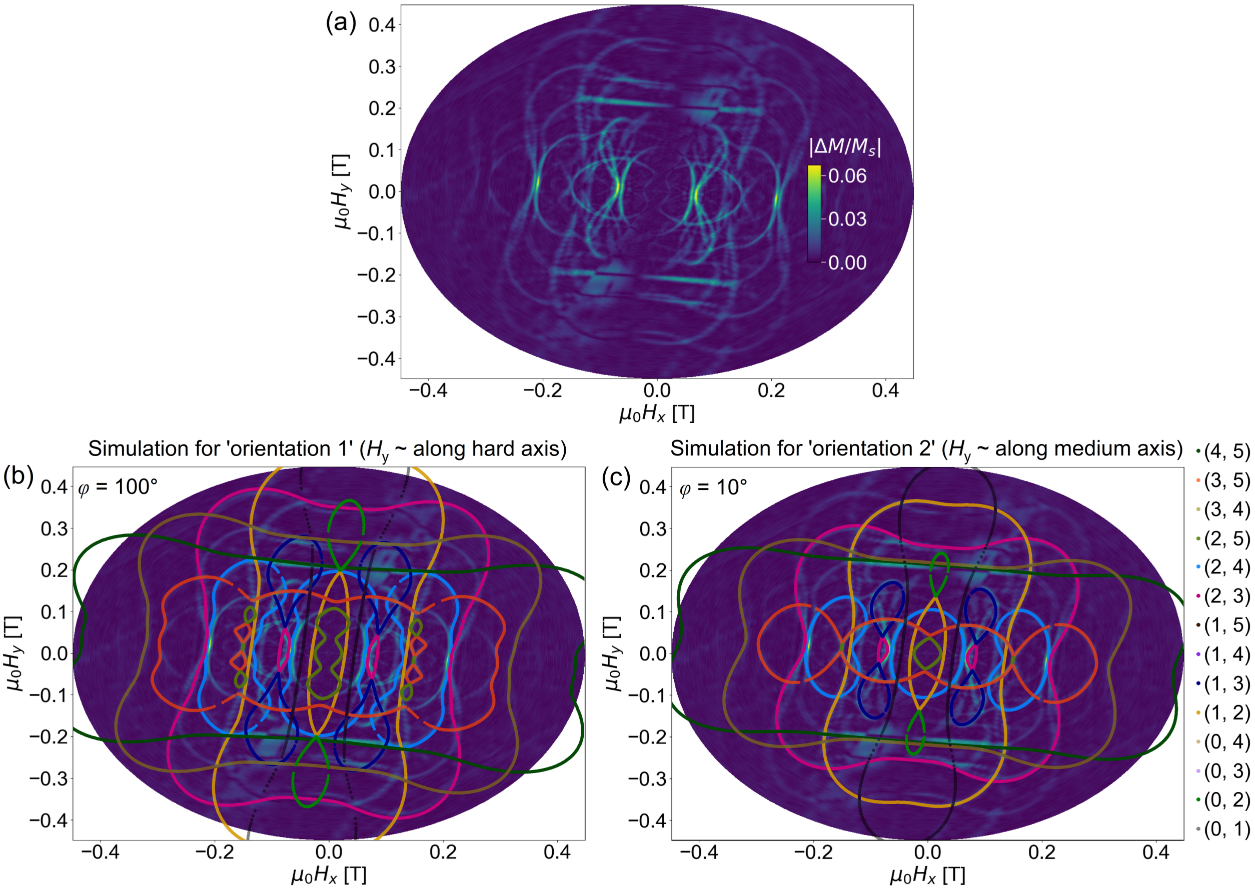

Figure S4. (a) Microwave absorption peak intensities as $|\Delta M/M_s|(H_{||})$ (after baseline subtraction) plotted as a function of applied field direction ($H_{x,y}$) at a fixed microwave frequency (8.90 GHz). The simulations in (b), (c) correspond to the two molecular orientations (see **Figure S1**) respectively. If H_y is along the hard axis for one orientation, it's inevitably along the medium axis for the other. Hence, (b) is simulated assuming H_y ~ nearly along the hard axis (φ ~ 100°); while (a) is simulated assuming H_y ~ nearly along the medium axis (φ ~ 10°). Any experimental feature in (a) can be found either in simulation (b) or in (c).

5. Animation of experimental frequency maps with transverse fields

The animation **SI.V1** shows several *frequency maps* (for $\boldsymbol{H}_{||}$, i.e. along the easy axis) at constant transverse field (H_{tr}) values stacked together to show how it evolves as $\boldsymbol{H}_{tr}$ is varied between -30 to 120 mT. As the $\boldsymbol{H}_{tr}$ is applied nearly along the hard axis of the system, the parity dependent oscillations in the avoided-level crossings (tunnel splittings) are observed. The animation compiles more than 72 hours of continuous data collection with a stable microwave, at a sweep rate for $\boldsymbol{H}_{||}$ <20 mT/s to

maintain adiabatic sweep, very small steps (0.5 mT) in the constant $\boldsymbol{H}_{tr}$ values. Each *frequency map* at a fixed $\boldsymbol{H}_{tr}$ contains 0.1 GHz steps in frequency, allowing precise visualization of the tunnel splittings.

6. Simulation of transverse field dependent Zeeman diagrams

To simulate these maps, the Zeeman diagrams need to be simulated in the presence of transverse magnetic fields. To apply constant transverse field (H_{tr}) using the 'levels' function in *EasySpin* simulations, the orientation ('theta'= θ, 'phi'=φ) of the crystal with respect to the applied field (H_z) was varied as a function of the longitudinal field. More specifically, θ, φ values were chosen as: $\theta = \tan^{-1}(H_{tr}/H_{||})$, and $\varphi = 0$ ($\pi/2$) for transverse field along the medium (hard) axis (and vice versa if the sign of B_2^2, B_4^4 are reversed).

In this way, the Zeeman diagrams are simulated (**Figure S5**) for different H_{tr} (at an angle φ = 100°) to compare with the experimental data. Notice that $\Delta_{1,2,3}$ do not oscillate in the same fashion, indicating the parity dependent features associated to QPI or topological quenching. Although here $\Delta_{2,3}$ oscillations cannot be clearly seen unless enlarged, it can be clearly understood from the fittings described in the main article. **Figure 4a** in the main article shows the fitted lines directly obtained by measuring $\Delta_{2,3}$ gaps from such Zeeman diagrams simulated at different H_{tr}.

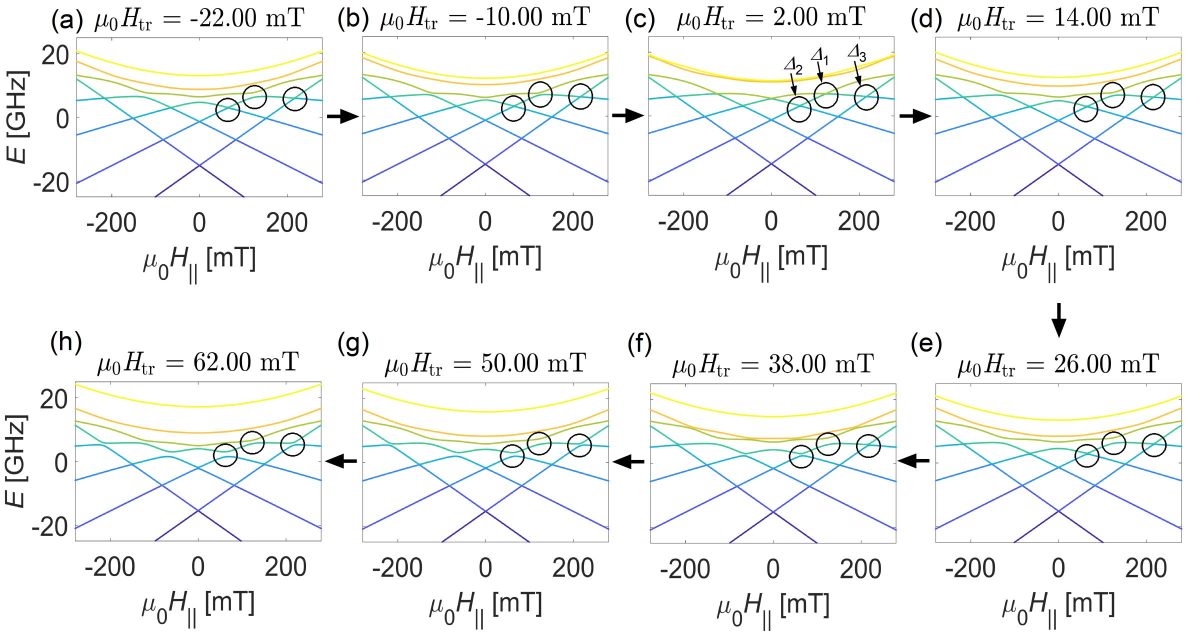

Figure S5. (a)-(h) Zeeman diagrams simulated for $H_{||}$ along the easy axis, with different constant values of transverse fields (H_{tr}) nearly along the hard axis (φ = 100°). Chosen H_{tr} values correspond to experimental Figure 2**.** The parity dependent oscillations in tunnel splittings ($\Delta_{1,2,3}$) can be observed. Notice that Δ_1 starts closing again from (f)-(h).

To show the complete set of simulations, the animation **SI. V2** stacks several Zeeman diagrams (for $H_{||}$, i.e. along the easy axis) at different H_{tr} values between -120 to +120 mT, with 1 mT steps is H_{tr} and at φ = 100°.

7. Angular dependence in $\boldsymbol{\Delta_{2,3}(H_{tr})}$ in hard-medium plane

Figure S6 shows $\Delta_2(\boldsymbol{H}_{tr})$ and $\Delta_3(\boldsymbol{H}_{tr})$ for two different cool-downs having different crystal orientations. In both cases the easy axis was aligned along $H_{||}$. In the first case, when $\boldsymbol{H}_{tr}$ is nearly aligned with the hard axis of the system (blue shades), oscillatory features are observed in $\Delta_{2,3}(\boldsymbol{H}_{tr})$, consistent with QPI effects. While in the second case, when $\boldsymbol{H}_{tr}$ is nearly aligned with the medium axis of the system (green shades), more classical-like monotonic increase in $\Delta_{2,3}(\boldsymbol{H}_{tr})$ is visible.

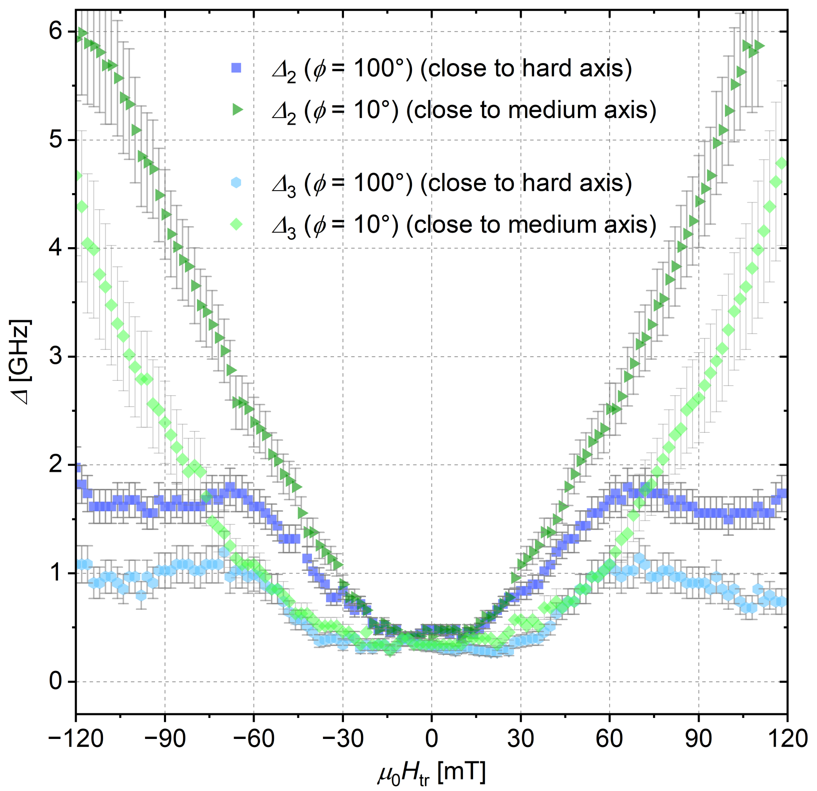

Figure S6. Experimentally obtained $\Delta_{2,3}(H_{tr})$ at two different angles of $\boldsymbol{H}_{tr}$ in the hard-medium plane obtained in two different cool-downs by rotating the crystal.

8. Classical energy diagram with 4th order transverse parameter

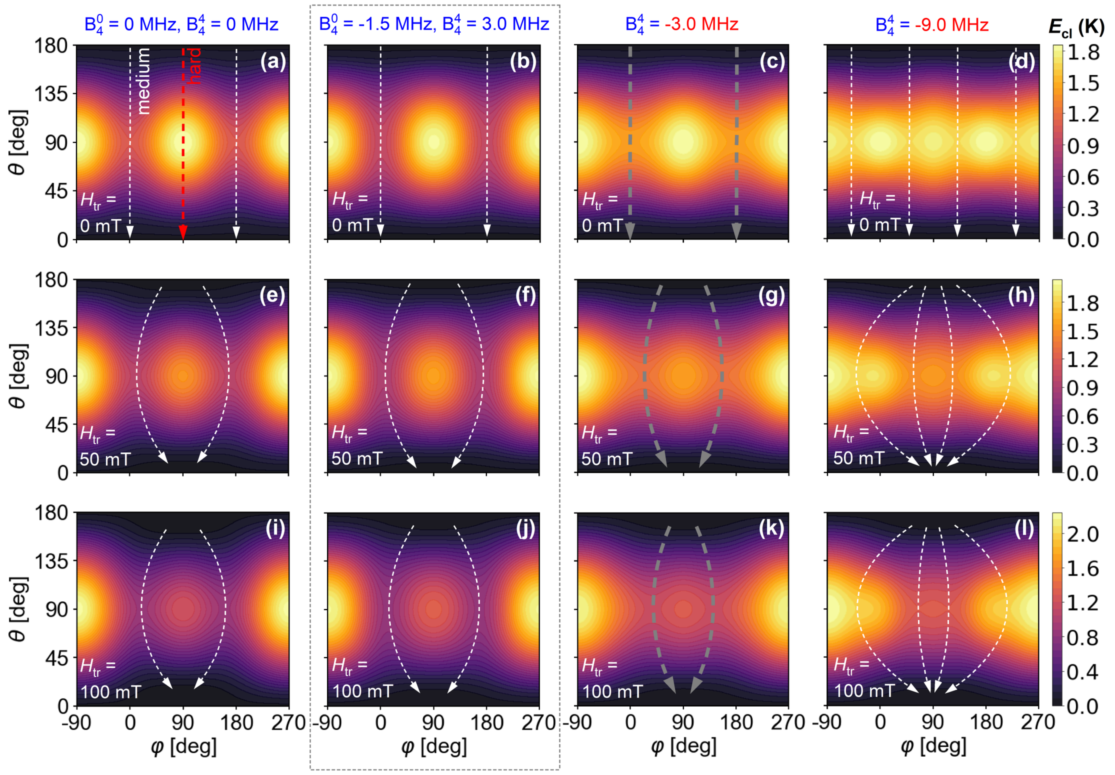

Figure S7: Classical energy E_{cl} mapped with classical spin orientation space (θ, φ) for the corresponding spin Hamiltonian (Eq. 1), with the ligand field parameters: $B_2^0 = -685\ MHz, B_2^2 = -275\ MHz$ and different values

for B_4^0, B_4^4 in absence of any external magnetic field ((a)-(d)). In these maps, θ = 0° and 180° (the poles) indicate the easy axis of the system, while θ = 90° line (equator) indicates the hard-medium plane with the hard axis at φ = 90° and medium at φ = 0°. The saddle points in the map E_{cl}(θ, φ) dictate the minimum energy pathway of magnetization reversal (tunneling) through the medium axis. As B_4^4 values are increased (with a same sign as B_2^2), the two interfering tunnel paths is found to split into 4 interfering paths. As the transverse magnetic field (H_{tr}) applied along the hard axis (φ = 90°) is increased ((e)-(l)), the two minima (easy axis) bends towards each other and away from the poles, and as a result, the area enclosed by the interfering paths monotonically decreases with increasing (H_{tr}). The dashed rectangular box indicates the transverse field dependent evolution of E_{cl} for the experimentally obtained ligand field parameter values (B_2^0, B_2^2, B_4^0 and B_4^4) in the $[GdPc_2]^-$ molecule.

A non-zero value of B_2^2, the coefficient of the 2nd order transverse term $O_2^2 \sim 1/2(S_+^2 + S_-^2)$, is a key ingredient to observe QPI in a spin system. This term, analogous to the transverse anisotropy coefficient E in the transition metal-based molecules, leads to anisotropy in the lateral plane, i.e. a hard and a medium axis. As a result, two preferred pathways (through the medium axis) dominate the quantum tunnelling rate between the two degenerate easy orientations. The QPI is therefore possible and can be detected as modulation of tunnel rate (QTM) by altering these two paths. For clarification and a qualitative overview of the QPI in the spin systems, a classical spin analogue (i.e. the magnetic moment $\vec{S}$ can orient freely in θ, φ space) is useful. **Figure S7** shows the (classical) energy landscape obtained from the above spin Hamiltonian by expanding the Stevens operators in terms of $\hat{S}_{x,y,z}$ and then writing them as continuous functions of θ, φ. The two (or more) interfering minimum energy pathways for quantum tunnelling and their evolution with transverse magnetic fields and different values of 4th order ligand field parameters are indicated. The parameters chosen for these simulations are neighbouring values to the experimentally obtained ligand field parameters in $[Et_4N]^{160}GdPc_2]$.

We can discuss the transverse field dependent oscillations in tunnel splittings from a qualitative point of view based on these energy diagrams. In the framework of time dependent Schrodinger's equation (with a time dependent Hamiltonian), at the diabolical points (DPs) the quantum mechanical wave function tunnels from one eigen state to the other. The most contributing tunnelling paths (as in **Figure S7**) lead to different geometric phases due to the intrinsic curvature in the parameter space. This additional phase difference (i.e. the Bery phase) leads to a term ~ cos($S.\Omega$) in the analytical expression for the tunnel splitting, where S is the spin and Ω is the area enclosed by the two interfering paths on the surface of unit sphere. As Ω in **Figure S7** monotonically reduces with increasing transverse fields, the tunnel rate exhibit oscillations as the signature of QPI (Berry phase) in the SMMs.

As further shown in **Figure S7**, the 4th order transverse terms, although apparently small, clearly play a significant role in magnetization dynamics by altering these QPI paths. A large value of B_4^4 can even result in four interfering paths (**Figure S7c**) instead of two. However, according to the details of QPI not entirely captured in the classical diagram, significant effect of B_4^4 starts to appear at much smaller values of it as described in the main article and the next section.

9. Non-trivial shifts of DPs with 4th order transverse parameter

Figure S8 shows the simulated variation of tunnel splittings $\Delta_{1,2}$ as a function of $\boldsymbol{H_{tr}}$ -direction in the hard-medium plane by plotting corresponding intersecting energy branches (at specific non-zero $H_{\parallel}$ values). Eventually the gaps between these intersecting levels, i.e. $\Delta_{1,2}$ are plotted as contour maps with $\boldsymbol{H_{tr}}$ -direction. Note that as B_4^4 varies (hypothetically), the DPs at Δ_1 do not change its position, however in the case of Δ_2, certain DPs move away from the hard-axis (towards the medium axis) depending on the sign of B_4^4 (with respect to the sign of B_2^2).

This sign-dependent effect caused by B_4^4 is intriguing, particularly because it can shift the DPs away from the hard axis, and moreover which of the DPs should undergo the major shift depends on the sign of B_4^4. As mentioned in the main article, this aspect needs to be investigated as a part of a broader theoretical study involving a perturbative approach, which we intend to publish elsewhere. Nonetheless, here we discuss one of the building blocks of this upcoming work with a logical demonstration how this B_4^4 sign-dependence is bound to arise when both B_2^2 and B_4^4 are present in the system.

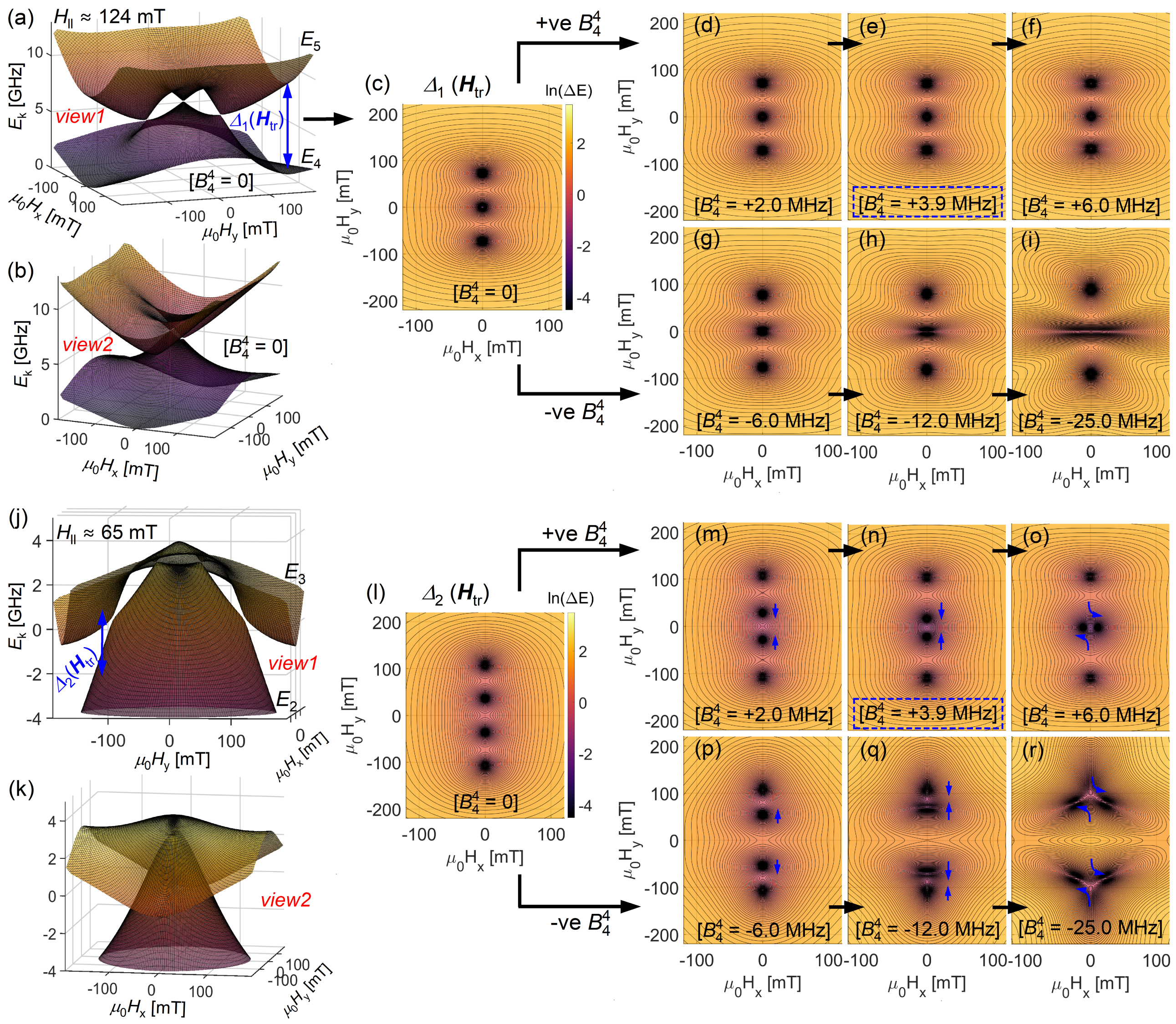

Figure S8: (a), (b) two different views of the simulated energy branches intersecting at Δ_1 (i.e. at $H_{||}$ ~ 124 mT), highlighting the three DPs in the orientation-space of $\boldsymbol{H}_{tr}$ applied in the hard-medium plane. The corresponding gap between the two intersecting levels is plotted in (c) as a contour map for a clear 2D view. The DPs clearly appear as dark dots as the gaps are plotted in the logarithmic scale. (a)-(c) are simulated for B_4^4= 0, while (d)-(i) shows the same for non-zero B_4^4. Panel (j), (k) show the two intersecting energy levels at Δ_2 (i.e. at $H_{||}$ ~ 65 mT), highlighting four DPs. The corresponding gaps between them, in (l)-(r) show the motion of DPs depending on sign of B_4^4.

To gradually introduce the effect of B_4^4, we discuss different cases of selectively setting transverse parameters to zero. First, if both B_2^2 and B_4^4 are zero, then there is complete isotropy in the xy plane, S_z is a good quantum number, and there is no possibility of any tunneling. In other words, all gaps are automatically zero, and we get perfect level crossings between curves with different S_z values at all possible locations. This case is a trivial one.

If B_4^4 = 0, but B_2^2 is non-zero, we get what may be called the "ideal Fe_8 DP pattern." The DP's fall on the perfect rhombic lattice as found by Keçecioğlu and Garg[10]. If B_2^2 =0, but B_4^4 is non-zero, we get the pattern described by Park and Garg[11]. These last two cases are elaborated in the following paragraphs in the context of the system we study here (S = 7/2).

For this discussion, let us choose the tunnel-gap between m_s: -7/2 and +7/2, defined here as Δ_0, and located at $H_{||}$ = 0 (the bottom-most tunnel-gap in the Zeeman diagram **Figure 1c** of the main article). This Δ_0 is not directly accessible in our experiments (probably plays a role in the 'tiling pattern' as discussed in the main article) due to low absorption peak intensity near zero-field and small

microwave frequencies. However, Δ_0 serves better to explain the effect in question, being more enriched (than $\Delta_{1,2}$ shown in **Figure S8**) and associated with 7 DPs as the transverse field is varied (see **Figure S9a**).

As depicted in **Figure S9a,** generally 7 DPs are associated with Δ_0, which lie equidistantly along the unique hard axis for 'Case I', i.e., $B_2^2 \neq 0$ and $B_4^4 = 0$. In 'Case IIa/IIb' ($B_2^2 = 0$ and $B_4^4 \neq 0$), due to the four-fold symmetric hard axes, the DPs are distributed in the medium-hard plane in the depicted way. In both *Case IIa* and *IIb*, there is a triply degenerate DP at $H_x = H_y = 0$. We note that, if $B_2^2 = 0$, a sign-flip of B_4^4 implies swapping between the 4-fold easy and hard axes, which leads to the DP pattern being rotated by 45° (see *Case IIa* and *IIb* in **Figure S9a**. Hence, in the absence B_2^2, the distinction between *Case IIa* and *IIb* is trivial.

However, if the Hamiltonian contains a dominant B_2^2 with a particular sign (that means a unique 'hard' and 'medium' axis), then the sign-dependent effect of B_4^4 becomes non-trivial. While tracking different DPs in our simulations, we confirmed that as the magnitude of B_4^4 is increased from zero, there is a sign-dependent pattern in the motion of DPs general to the tunnel-gaps with different Δm (as well as different spin systems: integer or half-integer). For $B_2^2 < 0$ and $B_4^4 > 0$ (opposite sign, i.e. the case studied in this work), as B_4^4 is gradually increased, the only viable way to reach from *Case I* to *Case IIa* is by the prior shift of inner DPs towards centre and then towards the 'medium' axis (see blue arrows in **Figure S9b**). In contrast, for $B_2^2 < 0$ and $B_4^4 < 0$ (same sign, as in ref. 50 in main article), the only viable way to reach from *Case I* to *Case IIb* with increasing B_4^4 is by the prior shift of outer DPs towards each-other and then towards 'medium' axis (see orange arrows in **Figure S9c**). These schematics provide a qualitative justification that the system studied here ($[GdPc_2]^-$: B_2^2 and B_4^4 with opposite sign) compliments the previous theoretical and experimental works (same sign) by allowing access to this experimentally unexplored regime and motivating further theoretical analysis. Note that, changing signs of B_2^2 and B_4^4 together simply implies a swapping between the x and y axis, hence **Figure S9b,c** suffices to explain all sign-combinations of these two transverse parameters.

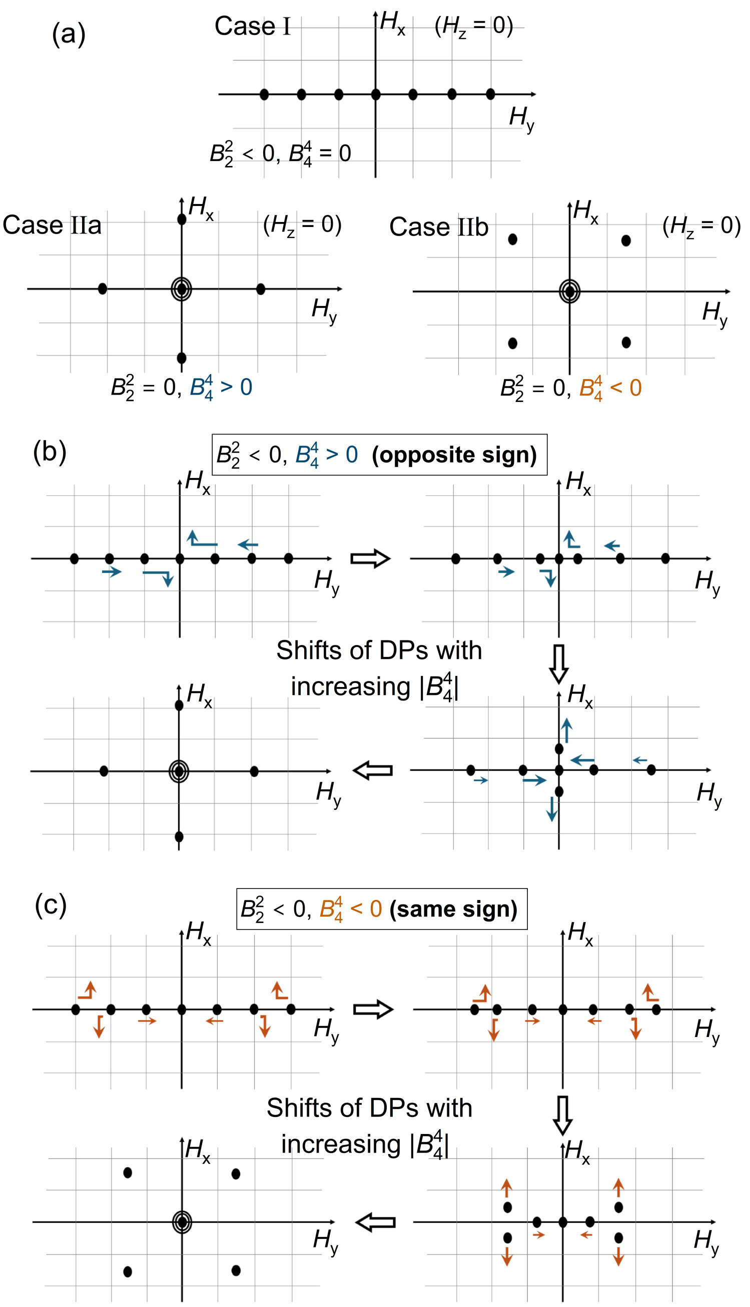

Figure S9: (a) Schematic representation of DPs associated to Δ_0 (at H_z = 0) for different hypothetical cases ($B_2^2 < 0$, $B_4^4 = 0$; $B_2^2 = 0$, $B_4^4 > 0$ and $B_2^2 = 0$, $B_4^4 < 0$). (b), (c) demonstrates how the DPs move (blue and red arrows) as $|B_4^4|$ is gradually increased from zero, to reach from 'Case I' to 'Case IIa' or 'IIb' respectively.

10. Reproduced results on another sample:

While the doubly constrained alignment criteria (both easy, hard axis desired in the measurement plane) limit the number of crystals for which the entire experiment can be repeated, we have very precisely reproduced the key observations on a second independent crystal/sample of the same system ($[GdPc_2]^-$), as shown in **Figure S10**. We note that the measurements in this 2nd sample are very precisely identical to that observed in the 1st sample studied thoroughly in our work, see **Figure S10(a),(b) and (c)** can be compared respectively with **Figure 1d** and **Figure 3a,b** (in main article).

The other general features of the frequency and angular maps when only the easy-axis was aligned in-plane were highly reproducible across crystals, both presented here and in earlier work by Taran et. al.[7], consistently yielding a unique parameter set except for the rotation-angle in the hard–medium plane.

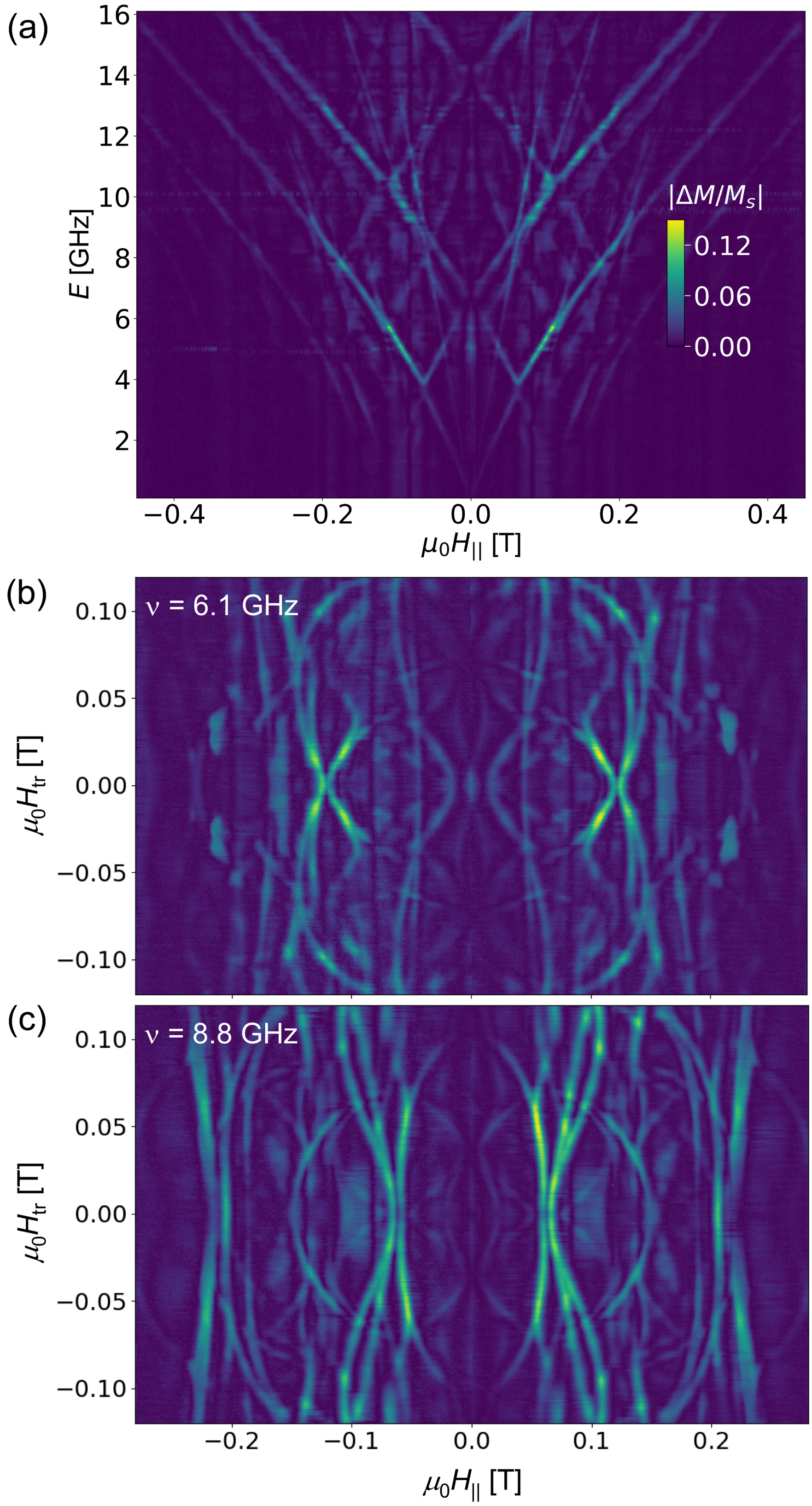

Figure S10: **Reproduced results on another sample:** Frequency map ($\Delta M(E, H_{||})$) along the easy axis (a). Transverse field dependent absorption maps ($\Delta M(H_{||}, H_{tr})$) between $\boldsymbol{H}_{tr}$ = ±120 mT at a fixed frequency of (b) 6.1 GHz and (c) 8.8 GHz. These figures: (a), (b), and (c) measured on a 2nd crystal of the same system ($[GdPc_2]^-$) can be compared respectively with **Figure 1d** and **Figure 3a,b** (in the main article) measured on the 1st sample thoroughly studied in this work.

Supplementary References

1 Fdez. Galván, I. *et al.* OpenMolcas: From Source Code to Insight. *Journal of Chemical Theory and Computation* **15**, 5925-5964 (2019). https://doi.org/10.1021/acs.jctc.9b00532

2 Aquilante, F. *et al.* Modern quantum chemistry with [Open]Molcas. *J. Chem. Phys.* **152**, 214117 (2020). https://doi.org/10.1063/5.0004835

3 Li Manni, G. *et al.* The OpenMolcas Web: A Community-Driven Approach to Advancing Computational Chemistry. *Journal of Chemical Theory and Computation* **19**, 6933-6991 (2023). https://doi.org/10.1021/acs.jctc.3c00182

4 Zobel, J. P., Widmark, P. O. & Veryazov, V. The ANO-R Basis Set. *J Chem Theory Comput* **16**, 278-294 (2020). https://doi.org/10.1021/acs.jctc.9b00873

5 Malmqvist, P. Å., Roos, B. O. & Schimmelpfennig, B. The restricted active space (RAS) state interaction approach with spin–orbit coupling. *Chem. Phys. Lett.* **357**, 230-240 (2002). https://doi.org/10.1016/s0009-2614(02)00498-0

6 Ungur, L. & Chibotaru, L. F. Ab Initio Crystal Field for Lanthanides. *Chem-Eur J* **23**, 3708-3718 (2017). https://doi.org/10.1002/chem.201605102

7 Taran, G. *et al.* Direct determination of high-order transverse ligand field parameters via µSQUID-EPR in a Et4N[160GdPc2] SMM. *Nat. Commun.* **14**, 3361 (2023). https://doi.org/10.1038/s41467-023-39003-5

8 Stoll, S. & Schweiger, A. EasySpin, a comprehensive software package for spectral simulation and analysis in EPR. *J Magn Reson* **178**, 42-55 (2006). https://doi.org/10.1016/j.jmr.2005.08.013

9 Stoll, S. & Britt, R. D. General and efficient simulation of pulse EPR spectra. *Phys Chem Chem Phys* **11**, 6614-6625 (2009). https://doi.org/10.1039/b907277b

10 Keçecioğlu, E. & Garg, A. Diabolical points in magnetic molecules: An exactly solvable model. *Phys. Rev. B* **63** (2001). https://doi.org/10.1103/PhysRevB.63.064422

11 Park, C.-S. & Garg, A. Topological quenching of spin tunneling in magnetic molecules with a fourfold easy axis. *Phys. Rev. B* **65** (2002). https://doi.org/10.1103/PhysRevB.65.064411
