# Supplementary material for: Topological suppression of quantum tunnelling in a lanthanide single-ion molecular magnet
Source: Nat Commun. 2026 Jun 26;17:5579. doi: 10.1038/s41467-026-74798-z (PMC13303870; doi:10.1038/s41467-026-74798-z)
Supplement: Supplementary file 2 — Supplementary Files [file 41467_2026_74798_MOESM2_ESM.pdf]

Description of Additional Supplementary Files

Supplementary Movie 1: Experimental frequency map upon varied transverse fields.

Supplementary Movie 2: Simulated Transverse field dependent Zeeman diagram.
