# Supplementary material for: Topological suppression of quantum tunnelling in a lanthanide single-ion molecular magnet
Source: Nat Commun. 2026 Jun 26;17:5579. doi: 10.1038/s41467-026-74798-z (PMC13303870; doi:10.1038/s41467-026-74798-z)
Supplement: Supplementary file 5 — Peer Review File [file 41467_2026_74798_MOESM5_ESM.pdf]

nature portfolio

Peer Review File

Topological Suppression of Quantum Tunnelling in a Lanthanide Single-Ion Molecular Magnet

Corresponding Author: Professor Wolfgang Wernsdorfer

This file contains all reviewer reports in order by version, followed by all author rebuttals in order by version.

Attachments originally included by the reviewers as part of their assessment can be found at the end of this file.

Version 0:

Reviewer comments:

Reviewer #1

(Remarks to the Author)
The manuscript by S. Paul et al is presenting a detailed study of Berry phase interference effect in a single-ion magnet. The experimental realization is complex and difficult which makes the results even more impressive. The authors used a micro-SQUID and a broadband waveguide to probe the spectroscopy of quantum levels, including the tunnel splittings of the molecular magnet.
The main article (MA) and the supplemental materials (SM) are well written, but they need some clarifications:
1. SM part 2: add a discussion about the micro-SQUID , mainly what material is made of, what is the substrate. How are the I+,I- lines used which are visible in Fig 1a? SQUIDs are made of superconducting (sc) material and the Ag waveguide is deposited on top. Does it mean that there is an electrical connection between sc and metal? Or is the sc oxidized? What is the thickness of the oxide? There is still a capacitive coupling between SQUID lines and the waveguide even if the SQUID is oxidized. Does that affect the SQUID switching pulse since it has a sharp rise? Is the electro-magnetic field penetrating the SQUID circuit via such capacitive coupling?
2. The frequency maps, such as Fig1d, show splittings located at 6-8 GHz. However, those energy levels are far above the ground state and the system is at 30mK. Discuss the thermal population aspect that allows measuring such excitations far from the ground state (MA or SM). If well visible, add arrows in Fig1c showing the measured transitions, or numbers on Fig1d (like 2->3).
3. MA line 129: perhaps the “very detailed” wording can be dropped
4. MA line 180. Give the numerical value of angle phi for the hard axis. This is done on line 243 so it can be deleted there and added at line 180 or so.
5. MA line 183: Typo, Figure 4b instead of Figure 4c
6. MA line 225: where is that equation coming from? Give reference, or explain it in SM.

Overall, I find the manuscript of high novelty and importance. I recommend it for publication once the minor points mentioned above are fixed.

Reviewer #2

(Remarks to the Author)
The manuscript by Sagar Paul et al. reports the application of a μSQUID-EPR magneto-spectroscopic technique to probe quantum phase interference (QPI) and topological quenching of quantum tunneling of magnetization (QTM) in Gd(III)-based molecular magnet $Et_4N[^{160}GdPc_2]^-$. The authors use resonant microwave absorption under variable longitudinal and transverse magnetic fields to map the full Zeeman diagram, resolve tunnel splittings (Δi) between specific ms states, and observe field-dependent oscillations in these splittings. These oscillations are interpreted as signatures of Berry phase interference, parity effects, and the influence of higher-order transverse anisotropy (particularly B44), leading to diabolical points (DPs) where tunneling gaps close due to destructive interference.

Overall, the manuscript is well-written, technically sound, and presents valuable experimental insights into QPI in 4f single-molecule magnets—an area where such direct, high-resolution mapping has been challenging. The results convincingly demonstrate the technique's power for precise spin-Hamiltonian parameter determination and controlled QTM quenching. I

recommend acceptance after the authors address the following major concerns and minor points.

i. The manuscript attributes the observed oscillations in tunnel splittings at specific transverse fields to "topological quenching" and "nontrivial topology." In many SMMs (4f systems), plotting spin energy levels against transverse field components will typically reveal avoided crossings and oscillating gaps. What specific criteria or additional evidence (beyond the observation of oscillations) distinguish these as truly topologically protected states, as opposed to conventional quantum interference? Are there any topological invariants, winding numbers, or other rigorous indicators that could be invoked? If not, the authors should temper claims of "nontrivial topology" to avoid potential overinterpretation, especially given the link to fault-tolerant quantum computation.

ii. Related to the above, the authors should clarify if it is appropriate to assume that the tunneling gaps found in Nature 531, 348–351 (2016) (atomic clock transitions for coherence enhancement) and Nature Physics 17, 1205–1209 (2021) (spin-electric control at clock transitions) exhibit analogous "topological" features under this definition? Overly broad application of topological terminology risks generating an excessive amount of papers that may mislead readers about the degree of protection or novelty relative to established interference effects. A brief discussion comparing/contrasting these clock transitions and other relevant references would strengthen the positioning of the present work.

iii. Berry phases are inherently gauge-invariant, but the manuscript's framing of the observed states as "topological" raises the question of whether the quenching/DPs are robust in a gauge-invariant sense (e.g., independent of specific gauge choices in the parameter space). If the topology is linked to the holonomy of the spin states under field trajectories, the authors could briefly address gauge invariance to reinforce the claims.

iv. The topological quenching effects are closely linked to the system's symmetry, particularly the transverse (off-diagonal) crystal-field parameters (B22 and B44) that enable multiple tunneling paths and Berry phase interference. To better elucidate the role of these terms in the claimed nontrivial topology, the authors should include numerical simulations (or at least discuss) where the off-diagonal parameters are selectively set to zero (or quenched) while keeping axial terms fixed and compare the resulting Zeeman diagrams, tunnel splittings, and diabolical points with the full model. This would directly demonstrate how symmetry breaking via transverse anisotropy drives the observed oscillations and gap quenching, strengthening the topological interpretation.

Minor Concerns/Questions
1. The conclusion mentions potential for "non-Abelian holonomies for HQC." While the work demonstrates Abelian Berry phases via QPI, non-Abelian holonomies typically require degenerate subspaces or multi-level control. A short clarification on whether the present results provide direct evidence for non-Abelian effects (or are aspirational) would be useful.

2. In SI Table S1, zero-field tunnel splittings appear (e.g., ~0.497 cm^{-1} for |±5/2⟩) alongside a highly anisotropic g-tensor (gx ≈ gy ≈ 0.05, gz ≈ 9.98 for that doublet; gx ≈ gy ≈ 8, gz ≈ 2 for |±1/2⟩). Could these residual gaps partly result from the non-axial g-anisotropy rather than solely from transverse crystal-field terms (B22, B44)? The authors might briefly clarify whether the fitted g-tensor contributes to these splittings or if they are fully attributed to ligand-field effects.

3. Did the authors record or estimate relaxation times (T_1/T_2) at the positions of Δ1, Δ2, and Δ3 (or near the diabolical points/minima)? Such data would help assess the practical coherence implications of the observed topological quenching and parity effects.

4. In the discussion of higher-order transverse effects, the sign of B44 (positive, opposite to B22) plays a key role in shifting diabolical points. A short remark on the chemical/symmetry intuition behind this sign (e.g., from the Pc_2 ligand environment) would add physical insight.

Reviewer #3

(Remarks to the Author)
see attached pdf

Version 1:

Reviewer comments:

Reviewer #1

(Remarks to the Author)
The authors modified the manuscript as needed; I recommend it for publication.

Reviewer #2

(Remarks to the Author)
I have carefully reviewed the authors’ responses to my comments and questions. I appreciate the authors’ efforts and thank

them for providing clear and satisfactory answers to my concerns. The revisions have improved the clarity and quality of the manuscript.

In its current form, I believe the manuscript is suitable for publication.

I therefore recommend acceptance.

Reviewer #3

(Remarks to the Author)
Dear Editor,

I have carefully reviewed the revised manuscript and the authors' point-by-point response to my original comments. Overall, the revision represents a substantial improvement, and the authors have addressed most of my concerns responsibly and in good faith.

Two residual concerns remain, which I flag for the editor's consideration rather than as blocking objections. First, regarding the comparison with Li et Garg (ref. 53) and the proposed sign-dependent role of B44: the authors acknowledge that a full perturbative treatment is deferred to a forthcoming theoretical publication. The discussion added to Section 9 of the SI and the numerical evidence from Figure S8 provide a reasonable basis for the claims made, and the authors are careful to frame the theoretical comparison as qualitative.
The manuscript language is now sufficiently hedged that I consider this acceptable for an experimental paper, provided the SI discussion is indeed as detailed as described in the rebuttal — I was not able to verify the revised SI directly, and I would ask the editor to confirm this. Second, regarding Figure S10: the rebuttal states that this figure reproduces the key features required for the analysis, including the transverse-field oscillations. If the oscillations in Delta(Htr) are clearly visible in the second-crystal data, reproducibility of the central observation is established; if only the easy-axis frequency map is shown, this point remains partially open.

Subject to editorial confirmation that the revised SI contains the transverse-field oscillation data for the second crystal and an adequately detailed Section 9, I recommend acceptance of the revised manuscript. The µSQUID-EPR methodology and the direct observation of Berry phase interference in a 4f molecular magnet represent a genuine experimental advance, and the paper is appropriate for publication in this journal.

Open Access This Peer Review File is licensed under a Creative Commons Attribution 4.0 International License, which permits use, sharing, adaptation, distribution and reproduction in any medium or format, as long as you give appropriate credit to the original author(s) and the source, provide a link to the Creative Commons license, and indicate if changes were made.
In cases where reviewers are anonymous, credit should be given to 'Anonymous Referee' and the source.
The images or other third party material in this Peer Review File are included in the article’s Creative Commons license, unless indicated otherwise in a credit line to the material. If material is not included in the article’s Creative Commons license and your intended use is not permitted by statutory regulation or exceeds the permitted use, you will need to obtain permission directly from the copyright holder.

To view a copy of this license, visit https://creativecommons.org/licenses/by/4.0/

Response to the Referees

On behalf of all co-authors, I would like to thank the referees for taking the time to carefully review our manuscript and for their valuable comments and suggestions, which undoubtedly will contribute to the betterment of the manuscript. We have followed all the comments and made revisions in the manuscript accordingly, which are highlighted in cyan. Our point-to-point responses to all questions and remarks are provided below.

Reviewer: 1

The manuscript by S. Paul et al. presents a detailed study of the Berry phase interference effect in a single-ion magnet. The experimental realization is complex and difficult, which makes the results even more impressive. The authors used a micro-SQUID and a broadband waveguide to probe the spectroscopy of quantum levels, including the tunnel splittings of the molecular magnet.

The main article (MA) and the supplemental materials (SM) are well written, but they need some clarifications:

Response: *We thank the referee for the suggestions. The raised points have been carefully considered and are now addressed in the revised manuscript and supplementary information.*

1. SM part 2: add a discussion about the micro-SQUID, mainly what material is made of, what is the substrate. How are the I+,I- lines used which are visible in Fig 1a? SQUIDs are made of superconducting (sc) material and the Ag waveguide is deposited on top. Does it mean that there is an electrical connection between sc and metal? Or is the sc oxidized? What is the thickness of the oxide? There is still a capacitive coupling between SQUID lines and the waveguide even if the SQUID is oxidized. Does that affect the SQUID switching pulse since it has a sharp rise? Is the electro-magnetic field penetrating the SQUID circuit via such capacitive coupling?

Response: *We have expanded the description of the device structure, operation, and clarified the raised concern about the capacitive coupling. As detailed in the updated supplemental information file (section 2), the μSQUIDs are fabricated from superconducting Nb film on a sapphire substrate. An AlOx spacer layer separates the superconducting SQUID circuitry from the Ag microwave waveguide deposited on top. This spacer ensures galvanic isolation between the superconducting circuitry and the metallic waveguide. The thickness of the AlOx layer is sufficient to prevent electrical contact, and no intentional conductive pathway exists between the SQUID and the waveguide. Standard μSQUID operation is performed using the I^+ and I^- bias lines, as illustrated in* ***Fig. 1a****. These current leads are used to bias the SQUID in a regime close to its critical current, allowing detection of switching events between the superconducting and resistive states.*

Regarding capacitive coupling: although the Nb SQUID and Ag waveguide are electrically isolated, a finite capacitive coupling is indeed expected due to their proximity. Such coupling may, in principle, allow electromagnetic fields from microwave pulses (particularly during fast switching edges) to weakly influence the μSQUID circuitry. For this reason, microwave irradiation and μSQUID readout are temporally separated in the experimental protocol, with a time window that is already significantly larger than the SQUID response time.

We have also verified experimentally that, in the microwave-off state, the critical current distributions of μSQUIDs located under the waveguide show only minor differences compared to reference devices without a waveguide. This indicates that any residual capacitive coupling does not significantly affect the SQUID operation under our measurement conditions.

Although sharp voltage or current edges could in principle generate transient electromagnetic coupling, the µSQUID measurement bandwidth is limited by the intrinsic dynamics of the Josephson junctions (including the Josephson characteristic time), which effectively filter very high-frequency perturbations. Consequently, the capacitive coupling does not measurably influence the switching measurements performed in this work. All these clarifications have now been added to the supplementary information file, section 2.

2. The frequency maps, such as Fig1d, show splittings located at 6-8 GHz. However, those energy levels are far above the ground state and the system is at 30mK. Discuss the thermal population aspect that allows measuring such excitations far from the ground state (MA or SM). If well visible, add arrows in Fig1c showing the measured transitions, or numbers on Fig1d (like 2->3).

Response: *The transitions observed in the 6–8 GHz range correspond to excited spin states that lie well above the ground state at the base temperature of 30 mK. We agree that clarification of the population mechanism is necessary.*

During microwave irradiation, the spin system is driven out of thermal equilibrium with the lattice. Under continuous microwave excitation, the spin populations are determined not solely by the cryostat temperature but by the balance between microwave pumping and spin–lattice relaxation processes. When the microwave frequency matches an energy level separation, resonant absorption redistributes the population among the involved spin states, enabling the detection of transitions between excited levels even if these lie far above the ground state at equilibrium.

In this regime, the effective spin population distribution deviates strongly from the Boltzmann distribution at 30 mK, allowing excited-state transitions such as those observed in the frequency maps. We have clarified this non-equilibrium aspect in the manuscript (page 3, after Eq. 1) to better explain how transitions in the 6–8 GHz range become experimentally accessible.

Additionally, the corresponding transitions are now explicitly labelled (e.g., (1,2) and (2,3)) in ***Fig. 1d*** *to improve clarity, as suggested by the referee.*

3. MA line 129: perhaps the “very detailed” wording can be dropped

Response: *The wording has been revised accordingly, and the expression “very detailed” has been removed from the manuscript.*

4. MA line 180. Give the numerical value of angle phi for the hard axis. This is done on line 243 so it can be deleted there and added at line 180 or so.

Response: *The numerical value of the hard-axis angle ϕ has now been added at the earlier location (line 180 in the previous version, page 5, last paragraph this version), as requested, to improve immediate clarity in the discussion.*

We have chosen to retain the additional mention at line 243 as well, since that section explains how the approximate value ϕ was determined. We believe keeping it there helps the reader follow the derivation and the reasoning behind its determination without needing to refer to earlier sections.

5. MA line 183: Typo, Figure 4b instead of Figure 4c

Response: *The typographical error has been corrected in the revised manuscript (the reference to Figure 4b has been amended to Figure 4c).*

6. MA line 225: Where is that equation coming from? Give reference, or explain it in SM.

Response: *The origin of the equation has now been clarified in the revised manuscript. A brief explanation of its derivation and physical basis has been added immediately following Eq. (2). In addition, a relevant reference has been included to properly support its use in the present context.*

Reviewer: 2

The manuscript by Sagar Paul et al. reports the application of a μSQUID-EPR magneto-spectroscopic technique to probe quantum phase interference (QPI) and topological quenching of quantum tunneling of magnetization (QTM) in Gd(III)-based molecular magnet Et4N[160GdPc2]⁻. The authors use resonant microwave absorption under variable longitudinal and transverse magnetic fields to map the full Zeeman diagram, resolve tunnel splittings (Δi) between specific ms states, and observe field-dependent oscillations in these splittings. These oscillations are interpreted as signatures of Berry phase interference, parity effects, and the influence of higher-order transverse anisotropy (particularly B44), leading to diabolical points (DPs) where tunneling gaps close due to destructive interference.

Overall, the manuscript is well-written, technically sound, and presents valuable experimental insights into QPI in 4f single-molecule magnets—an area where such direct, high-resolution mapping has been challenging. The results convincingly demonstrate the technique's power for precise spin-Hamiltonian parameter determination and controlled QTM quenching. I recommend acceptance after the authors address the following major concerns and minor points.

Response: *We thank the reviewer for the insightful comments and valuable suggestions. Several of the points raised prompted us to examine the underlying questions in greater depth, leading to a clearer articulation of the key theoretical aspects of the work in the revised manuscript.*

1. The manuscript attributes the observed oscillations in tunnel splittings at specific transverse fields to "topological quenching" and "nontrivial topology." In many SMMs (4f systems), plotting spin energy levels against transverse field components will typically reveal avoided crossings and oscillating gaps. What specific criteria or additional evidence (beyond the observation of oscillations) distinguish these as truly topologically protected states, as opposed to conventional quantum interference? Are there any topological invariants, winding numbers, or other rigorous indicators that could be invoked? If not, the authors should temper claims of "nontrivial topology" to avoid potential overinterpretation, especially given the link to fault-tolerant quantum computation.

Response: *We agree with the referee that gap oscillations alone do not constitute ironclad evidence for the claim that they are topological. There are two ways in which one could proceed, reflecting different aspects of the word topological. The first, most desirable, and experimental way would be to show that the pattern (not the precise locations) of diabolical points (DP's) is robust against small changes in the material parameters (B_2^0, B_2^2, B_4^4 etc.). Unfortunately, this is a nearly impossible bar because it would require making completely new, chemically different samples. The second way is numerical, following the criteria proposed by Berry and subsequently implemented in other studies of single-molecule magnets (Phys. Rev. B 65, 064411). This way allows us to confirm that the observed oscillations indeed arise from true DP's, and puts the entire data-set in the framework and language of topological quenching, DP's, Berry phases, etc. We note in passing that this is the only successful framework to date for directly explaining the gap oscillations in any SMM*

Thus, we evaluate the Berry-phase acquired by the two energy levels near a degeneracy under a circuit or loop in the transverse magnetic field. The accumulated phase is evaluated using a Python script (see 10.5281/zenodo.19451754), which numerically diagonalizes the Hamiltonian

and computes the phase associated with the selected eigenstate along a closed circular trajectory in parameter space. The accumulated phase is π for loops encircling a DP, while 0 loops do not encircle a DP. This quantized π/0 behaviour is the special Longuet-Higgins (ref 47 in the main MS) subcase of the Berry phase and is the topological invariant that the referee is asking for.

*In particular, referring to **Fig. 4a**, all the gap minima for Δ_2 and Δ_3, and that for Δ_1 near $\mu_0 H_{tr}$ = 65 mT, are DP's that cannot be understood as Wigner-von Neumann symmetry-allowed level crossings. Experimentally, of course, we cannot see gap zeros, only minima, due to imperfect alignment and effective stray fields with multiple origins.*

We now mention these numerics and arguments in the updated main manuscript (page 6: paragraph 1 and page 7: 4th paragraph of section "Numerical simulation of fourth-order transverse parameter effects & Berry phase"). While the additional script (see 10.5281/zenodo.19451754) and accompanying discussion fully support our conclusions regarding 'topological quenching', we have, in accordance with the referee's recommendation, removed the term 'non-trivial' topology for the sake of simplicity.

2. Related to the above, the authors should clarify if it is appropriate to assume that the tunneling gaps found in Nature 531, 348–351 (2016) (atomic clock transitions for coherence enhancement) and Nature Physics 17, 1205–1209 (2021) (spin-electric control at clock transitions) exhibit analogous "topological" features under this definition? Overly broad application of topological terminology risks generating an excessive amount of papers that may mislead readers about the degree of protection or novelty relative to established interference effects. A brief discussion comparing/contrasting these clock transitions and other relevant references would strengthen the positioning of the present work.

***Response:** We agree that a discussion can be added to compare/distinguish the observed avoided crossings in clock transitions in the context of the discussed DPs in this work.*

Clock transitions arise at points where the transition frequency becomes first-order insensitive to magnetic-field fluctuations ($\partial\omega/\partial B \approx 0$), thereby enhancing coherence by suppressing dephasing. This effect also originates from the structure of the spin Hamiltonian and level mixing, but may not directly rely on a topological invariant property. However, this region (large and flat tunnel gaps) can be discussed in the terminology of Diabolical Points (DPs).

The oscillatory suppression of tunnel splittings (DPs) observed in the present work originates from Berry-phase interference between symmetry-related tunnelling trajectories. While both phenomena (DPs and clock transitions) involve special points in the field-dependent spectrum, their physical origin can be different: clock transitions correspond to a large tunnel gap, which might be a maximum of the tunnel gaps if plotted with transverse fields, whereas Berry-phase interference produces quenching (or DPs, i.e., the minima) of tunnelling gaps through destructive phase accumulation. Whether the tunnel-gap in clock transition lies exactly between two DPs (i.e., a maximum of tunnel gap) or not will depend on its transverse-field- (or transverse-parameter-) dependent investigation. Although we did not identify prior studies on this point, we expect that examining the clock-transition systems in this context would be valuable, particularly for determining the role of the Berry phase in shaping the clock-transition gap.

Hence, we have included a brief comparative discussion on clock transitions in the context of DPs, referencing the works cited by the reviewer (page 7 of main manuscript: last paragraph before the section: "Numerical simulation….").

3. Berry phases are inherently gauge-invariant, but the manuscript's framing of the observed states as "topological" raises the question of whether the quenching/DPs are robust in a gauge-invariant sense (e.g., independent of specific gauge choices in the parameter space). If the topology is linked to the holonomy of the spin states under field trajectories, the authors could briefly address gauge invariance to reinforce the claims.

Response: *The referee is again correct about the gauge invariance of Berry phases. However, gauge invariance and the topological aspects go hand in hand and cannot really be separated. Gauge-invariance is implicit in the numerical loop calculations described in our answer to Q1. Accordingly, the raised concern is addressed in the updated main manuscript in the paragraph describing the numerical evaluation of Berry phase and mentioning the words 'topological invariant' (page 6: paragraph 1 and page 7: 4th paragraph of section "Numerical simulation of fourth-order transverse parameter effects & Berry phase"). In this context, noting 'gauge-invariant' is not essential anymore, as topological invariants are inherently gauge-invariant.*

4. The topological quenching effects are closely linked to the system's symmetry, particularly the transverse (off-diagonal) crystal-field parameters (B22 and B44) that enable multiple tunneling paths and Berry phase interference. To better elucidate the role of these terms in the claimed nontrivial topology, the authors should include numerical simulations (or at least discuss) where the off-diagonal parameters are selectively set to zero (or quenched) while keeping axial terms fixed and compare the resulting Zeeman diagrams, tunnel splittings, and diabolical points with the full model. This would directly demonstrate how symmetry breaking via transverse anisotropy drives the observed oscillations and gap quenching, strengthening the topological interpretation.

Response: *We have indeed done exactly the simulations the referee is asking for, but we are reluctant to discuss them too much in this paper, given that they fall more into a theoretical work, which will be part of a larger theoretical analysis we intend to publish separately as a standalone theoretical paper. To focus too much on them would take away from the experimental thrust of this paper. Nevertheless, we have provided a partial discussion (that will be elaborated in our later theoretical work) in the updated section 9 of the SI, as mentioned briefly on page 7 (2nd paragraph) in the main manuscript. This discussion also includes the effect of selectively setting the off-diagonal terms to zero.*

5. The conclusion mentions potential for "non-Abelian holonomies for HQC." While the work demonstrates Abelian Berry phases via QPI, non-Abelian holonomies typically require degenerate subspaces or multi-level control. A short clarification on whether the present results provide direct evidence for non-Abelian effects (or are aspirational) would be useful.

Response: *The present work demonstrates oscillatory suppression of tunnel splittings arising from Berry-phase interference and is probably too limited to distinguish Abelian vs non-Abelian geometric phase. We agree that non-Abelian holonomies generally require adiabatic evolution within a degenerate multi-level subspace, leading to matrix-valued geometric phases. The results presented here do not provide direct evidence for such non-Abelian effects; however, this possibility cannot be completely overlooked, as in the present systems, there exist DPs with exactly zero tunnel gaps (at least theoretically or in simulation).*

Our intention in mentioning non-Abelian holonomies was to highlight a possible long-term perspective (hence purely aspirational): systems exhibiting controllable degeneracy points and geometric phase effects may, in principle, provide a platform for engineering more complex holonomic operations. However, we agree that this could be interpreted as an overstatement.

We have therefore revised the conclusion to clarify that the present results are too limited to differentiate or clarify what to expect in Abelian and non-Abelian cases, and that any connection to non-Abelian holonomies should be regarded as a prospective direction rather than a demonstrated outcome (see revised 'Conclusion' in page 9).

6. In SI Table S1, zero-field tunnel splittings appear (e.g., ~0.497 cm^{-1} for |±5/2⟩) alongside a highly anisotropic g-tensor ($g_x \approx g_y \approx 0.05$, $g_z \approx 9.98$ for that doublet; $g_x \approx g_y \approx 8$, $g_z \approx 2$ for |±1/2⟩). Could these residual gaps partly result from the non-axial g-anisotropy rather

than solely from transverse crystal-field terms (B22, B44)? The authors might briefly clarify whether the fitted g-tensor contributes to these splittings or if they are fully attributed to ligand-field effects.

Response: *Table S1 reports the zero-field splittings (ZFS) of the Kramers doublets (i.e., the energy separation between the eigenstates of each doublet), not the tunnel splittings. These zero-field splittings (such as the energy separation between |7/2⟩ and |5/2⟩ doublets) arise exclusively from the axial ligand-field parameters included in the Hamiltonian (e.g., B_2^0 B_4^0, etc.), while tunnel-splittings (such as between |+7/2⟩ and |-7/2⟩) depend on both axial and transverse parameters, and Berry-phase. The g-tensors listed in the table reflect the anisotropy imposed on each doublet, but given zero longitudinal and transverse (H_x, H_y = 0) fields, the g_x, g_y cannot replicate a tunnel-gap (at zero-field) as the associated Zeeman terms (~$g_x H_x$) are zero.*

7. Did the authors record or estimate relaxation times (T_1/T_2) at the positions of Δ1, Δ2, and Δ3 (or near the diabolical points/minima)? Such data would help assess the practical coherence implications of the observed topological quenching and parity effects.

Response: *We appreciate the referee's insightful suggestion. Indeed, measuring or estimating T_1 and T_2 at the positions of the Δ_1, Δ_2, and Δ_3 gaps would be extremely valuable for assessing the coherence implications of the observed topological quenching and parity-protected crossings. However, obtaining such data requires a different experimental methodology, namely pulsed EPR (with pulse sizes much smaller and/or delay much larger than what is used, and preferably with a pulse train design as in echo detected EPR). At present, we are conducting parallel efforts using such pulsed-EPR to probe relaxation processes near these avoided crossings. While we have obtained preliminary signals, we are not yet able to reliably determine T_1 at the relevant gaps. This arises due to sensitivity*

At millikelvin temperatures (~30 mK), the excited Kramers doublets remain essentially unpopulated, making transitions involving Δ_2 and Δ_3 inaccessible without raising the bath temperature—something that would compromise μSQUID sensitivity. Even the nominally accessible 0→1 transition becomes too weak to detect near zero field due to the very low microwave frequencies required. Furthermore, when following the 0→1 transition toward zero field, the system crosses the 1→2 gap around 0.1 T, but this excitation pathway does not probe the 1→2 dynamics, preventing relaxation measurements at Δ_2.

1. ***Accessing the excited-state transitions at millikelvin temperatures***
   Even with pulsed techniques, the spin temperature at ~ 30 mK remains too low to significantly populate the excited Kramers doublets. Without raising the bath temperature to several kelvins—currently beyond the scope as the μSQUID sensitivity for such intricate details becomes marginal at elevated bath temperatures—many of the transitions associated with Δ_2 and Δ_3 cannot be accessed with sufficient sensitivity.
2. ***Challenges at zero field and low microwave frequencies***
   Among the accessible gaps, the one at zero field (following the $0 \rightarrow 1$ transition down to $B = 0$) is, in principle, measurable. However, the microwave absorption becomes extremely weak near zero field and at the very low microwave frequencies required to excite this transition, making T_1 extraction impractical with the current instrumentation.
3. ***Crossing of higher-lying gaps without excitation of the associated transitions***
   When tracking the $0 \rightarrow 1$ transition towards zero field, the system passes through the crossing associated with the $1 \rightarrow 2$ gap at approximately 0.1 T. Nonetheless, the $0 \rightarrow 1$ excitation does not effectively probe the dynamics of the $1 \rightarrow 2$ transition at that point, preventing a direct measurement of relaxation at Δ_2.

In summary, while we fully agree on the scientific value of such measurements, the combination of population constraints, weak absorption at ultra-low fields/frequencies, and the

need for different experimental transitions currently prevents us from extracting reliable T_1/ T_2 values at the diabolical points. We hope that forthcoming advances in instrumentation will make these measurements feasible in the future.

8. In the discussion of higher-order transverse effects, the sign of B44 (positive, opposite to B22) plays a key role in shifting diabolical points. A short remark on the chemical/symmetry intuition behind this sign (e.g., from the Pc_2 ligand environment) would add physical insight.

Response: *We agree that providing some physical intuition for the sign of the transverse crystal-field parameters can help clarify their role in the behaviour of the tunnel splittings and the position of the diabolical points.*

In lanthanide complexes, the sign and magnitude of higher-order transverse crystal-field parameters such as B_4^4 are determined by the symmetry and spatial distribution of the surrounding ligand field. In the present system, the Pc_2 coordination environment imposes a pronounced fourfold component to the electrostatic potential in the equatorial plane. This symmetry naturally gives rise to fourth-order transverse crystal-field terms, whose sign reflects the orientation of the ligand donor atoms relative to the principal magnetic axes. In this framework, the positive B_4^4 term (opposite in sign to B_2^2) modifies the transverse anisotropy landscape and therefore shifts the positions of the diabolical points associated with Berry-phase interference. Note that the presence of the B_2^2 term arises due to symmetry lowering of the $[GdPc_2]^-$, as also observed in the archetypal $[Mn_{12}(\mu_3\text{-}O_{12})(CH_3COO)_{16}(H_2O)_4]\cdot 2CH_3COOH\cdot 2H_2O$ $\{Mn_{12}\}$ system (ref. 44 in the main MS).

While a detailed microscopic derivation of the crystal-field parameters would require a full ligand-field analysis, the observed signs of B_2^2 and B_4^4 are consistent with the symmetry of the Pc_2 ligand environment and the resulting fourfold modulation of the transverse anisotropy.

Following the referee's suggestion, we have added a short remark in the manuscript to clarify the chemical and symmetry-related origin of this parameter and its influence on the position of the diabolical points (page 8, and in section 9 of the Supplementary Information file).

Reviewer: 3

This manuscript reports the direct spectroscopic observation of quantum phase interference (QPI) and parity-dependent oscillations in tunnel splittings in a gadolinium-based single-ion molecular magnet, [160GdPc2]−. The authors employ a combined μSQUID-EPR technique to map the complete spin manifold (S = 7/2) under transverse magnetic fields, resolving avoided level crossings that reveal topologically quenched quantum tunnelling of magnetisation (QTM). The work claims to demonstrate the first direct observation of Berry phase effects in a 4f-based molecular magnet and highlights the significant role of fourth-order transverse ligand field parameters (B44) in determining the positions of diabolical points.

The manuscript addresses an important gap in the field of molecular magnetism. While QPI has been observed in 3d-based molecular magnets (Fe8, Mn12) through Landau-Zener tunnelling experiments, extending these observations to lanthanide systems has remained challenging due to the larger tunnel gaps that preclude conventional approaches. The μSQUID-EPR methodology presented here represents a genuine technical advance that enables direct spectroscopic access to tunnel splittings in 4f systems.

If the claims are validated, this work would constitute a significant contribution with implications for understanding spin topology in lanthanide-based quantum systems, rational design of molecular qubits with controlled tunnelling rates, and development of holonomic quantum computation schemes using molecular magnets.

However, several critical issues must be addressed before publication can be recommended.

Response: *We sincerely thank the referee for the careful reading of our manuscript and the thoughtful remarks provided. The observations offered clarified several points and guided us toward a more precise treatment of uncertainties and a more careful analysis of the data.*

1. Significant Discrepancy Between CASSCF Calculations and Experimental Parameters: This is the most significant concern with the manuscript. Table S2 in the Supplementary Information reports crystal field parameters extracted from CASSCF-SO calculations, while the main text provides experimentally determined values. A careful comparison reveals substantial inconsistencies. For the axial parameters, the agreement is reasonable: the CASSCF value of B02 = −811 MHz compares to the experimental value of −685 MHz (ratio ~ 1.2), and B04 = −0.239 MHz from CASSCF compares to −1.45 MHz experimentally (ratio ~ 6). However, for the transverse parameters, the discrepancies are far more severe. The CASSCF calculation yields B22 = 3.73×10−2 MHz, whereas the experimental fitting requires B22 = −275 MHz. This represents a difference of approximately four orders of magnitude (~ 7400×). Similarly, direction of the transverse field relative to the molecular axes, the two orientations should exhibit qualitatively different behaviour.

Response: *We agree that it's crucial to discuss the discrepancy between the crystal-field parameters obtained from CASSCF-SO calculations (Table S2 in the Supporting Information) and those extracted from the experimental analysis. While the axial parameters show agreement within at least one order of magnitude, the transverse parameter B_2^2 differs substantially, and this point requires clarification.*

First, we emphasize that the CASSCF calculations are performed on an isolated molecule based on the crystallographic structure, and therefore describe an idealized, near-symmetric environment (approximately D_{4d}/C_4). In such a high-symmetry setting, the crystal-field Hamiltonian is strongly constrained, and second-order transverse terms such as $B_2^2$are expected to be very small or even symmetry-forbidden in the strict limit. The computed value small B_2^2 is therefore consistent with a nearly ideal symmetry.

Across a broad range of systems, deviations from CASSCF-predicted anisotropy parameters are commonly observed, including also for the axial (dominant) components. Such deviations typically retain the overall order-of-magnitude and sign but differ quantitatively from the ab-initio values. Accordingly, departures from CASSCF results in the axial parameters of the present system are consistent with this general behaviour. To try to account for lower symmetry, an extended calculation, comprising the Et_4N^+ counteraction was carried out, however, the difference in experimental and calculated transverse parameters, particularly B_2^2 remains large, hence this needs to be elaborated.

In contrast to the CASSCF simulations in the idealized environment, the experimentally fitted parameters correspond to the effective crystal-field Hamiltonian in the solid-state environment, where several additional effects come into play. These include intermolecular electrostatic interactions, crystal packing, structural distortions, and possible symmetry lowering induced by substitutional disorder (Y → Gd), chemical disorder. Even modest deviations from ideal symmetry can introduce and significantly change transverse crystal-field terms that are otherwise suppressed. We have theoretically investigated such a possibility by applying an elliptical distortion to one of the Pc ligands of the $Et_4N[GdPc_2]$ complex and computed the CASSCF electronic properties. The results show that minor distortions (1-3%) can lead to rather large changes in the B22 parameter, which is a direct result of symmetry lowering (See ***Figure SR. 1****).*

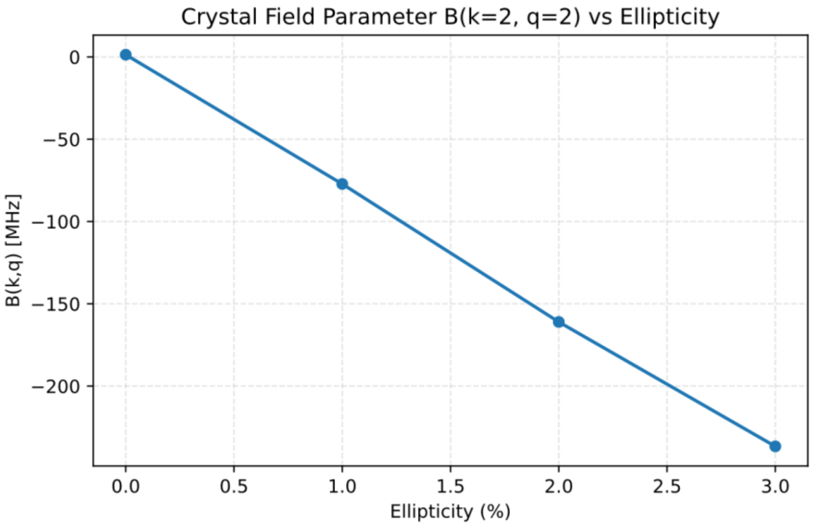

Figure SR1. *B_2^2 vs hypothetical elliptic distortion on one of the Pc ligands comprising the $Et_4N[GdPc_2]$ complex.*

Such symmetry-lowering effects are particularly important at very low temperatures (down to ~30 mK), as the crystal structure may undergo small (gradual or abrupt) temperature-dependent deviations as the system is cooled from room-temperature to low temperature. Cooling the system from room temperature to low temperature can introduce gradual (or even abrupt) structural changes, and at sub-Kelvin temperatures, the system is likely frozen into a configuration that departs (as observed in several measurements of the same system) from its room-temperature structure (XRD), as discussed earlier. Such a behaviour has also been observed in the archetypal $\{Mn_{12}\}$ with 4-fold symmetry (ref. 44 in the main MS).

Moreover, we have also investigated the possibility that the µSQUID-EPR maps to originate from a D_{4d}/C_4 symmetry. In the perspective of the Stevens operator formalism, this difference is physically meaningful. The operator

$$\hat{O}_2^2 = \frac{1}{2}(S_+^2 + S_-^2)$$

introduces mixing between states with $\Delta m = \pm 2$, thereby opening tunnelling channels that are essential to reproduce the experimentally observed transitions. In contrast, in strict D_{4d}/C_4symmetry the transverse anisotropy is dominated by higher-order terms such as $B_4^4\hat{O}_4^4$, which couple states with $\Delta m = \pm 4$ and generate a qualitatively different mixing pattern.

Indeed, during the fitting procedure we verified that restricting the Hamiltonian to symmetry-allowed terms (i.e., excluding B_2^2) fails to reproduce several key experimental features. The inclusion of a finite B_2^2 term is therefore necessary, indicating that the effective symmetry experienced by the Gd ion is lower than the idealized molecular symmetry.

Finally, regarding the sign difference of B_2^2, we note that its sign depends on the orientation of the magnetic axes relative to the ligand field and can change under small rotations of the coordinate system or symmetry lowering. The experimentally determined sign therefore reflects the actual anisotropy in the crystal environment, rather than the idealized frame used in the ab initio calculation.

Table S2 has been updated with the newly calculated CASSCF parameters, considering the counter cation, while a sentence regarding the distortion effect leading to symmetry lowering has been added to the MS on page 3.

2. How do the authors distinguish or separate contributions from the two molecular orientations in their µSQUID-EPR data?

Response: *We appreciate the reviewer's observation on the two molecular orientations which motivated us to discuss it the updated manuscript. We find the presence of two molecular orientations in the (5%) diluted crystal as a useful aspect, as it benefitted the analysis rather than complicating it.*

*The two crystallographic sites are non-interacting as the 5% diluted crystal contains mostly non-magnetic $[YPc_2]^-$ surrounding isolated $[GdPc_2]^-$ sites. However, as the applied $\mathbf{H}_{tr}$ aligns with the hard axis of one site, it is inevitably aligned along the medium axis of the other (see the symmetries imposed in **Figure S1**). As both the (non-interacting) sites compose the system, for every measurement the information contains both orientations.*

*Nevertheless, when the transverse field is aligned along the hard axis of one molecular orientation (and therefore simultaneously along the medium axis of the other), the behavior of the two sites becomes unmistakably distinct: (1) One site exhibits the characteristic oscillatory modulation of the tunnel gap, while (2) the other displays a monotonic increase (divergence) of Δ. Additionally, note that the QPI features (at each tunnel-gap) for different angles in the hard-medium plane do not cross each other (see the simulations in **Figure 4d**). These aspects make the extraction of tunnel gaps feasible with the two molecular orientations clearly distinguished for most of the measured orientations.*

Eventually, rather than being a disadvantage, the existence of two well-separated angular behaviours offered additional constraint to uniquely determine/ confirm the direction of $\mathbf{H}_{tr}$ with respect to the individual molecular frames. This is now discussed in the updated main manuscript (page 5, the last paragraph before the Section: "Topological quenching and parity effect").

3. When mapping Δi(Htr) oscillations, do both orientations contribute simultaneously? If so, how is this deconvoluted?

Response: *As noted above, both molecular orientations contribute simultaneously when mapping the $\Delta_i(H_{tr})$ oscillations. However, their contributions are distinguishable for the reasons elaborated above in the previous answer.*

Because the two crystallographic sites are non-interacting and their tunnel-splitting oscillations do not cross as the transverse field is rotated in the hard–medium plane, each site exhibits a characteristic and easily distinguishable behaviour as now discussed in the updated main manuscript (page 5, the last paragraph before the Section: "Topological quenching and parity effect").

4. Could the observed "tiling pattern" near H‖ = 0 (Figures 3a,b) arise partially from superposition of signals from the two orientations rather than purely from QPI effects?

Response: *As discussed above, both molecular orientations contribute to the μSQUID-EPR signal near $H_{||}$ =0. However, their behaviours are qualitatively distinct for a given direction of $\mathbf{H}_{tr}$: the orientation with $\mathbf{H}_{tr}$ along its hard axis produces the characteristic oscillatory QPI pattern (the "tiling"), whereas the orientation with $\mathbf{H}_{tr}$ nearly along its medium axis yields only a smooth, monotonic increase in the tunnel gap. Because these trends are largely non-overlapping and the sites are non-interacting, their contributions are distinguishable. The observed tiling pattern originates from QPI of the hard-axis-aligned orientation, with the second orientation contributing only a smoothly expanding pattern. The tiling patterns in the experimental **Figure 3a** agree with the simulations in **Figure 3c**, which consider only one molecular orientation, supporting that these features originate solely from QPI effects. The additional structures present in Figure 3a but absent from **Figure 3c** arise predominantly from the second molecular orientation.*

5. The fitting of angular maps (Figure S4) should show evidence of two sets of transitions if both orientations *contribute. Is this observed?*

Response: *Yes. The updated angular-map fittings (Supporting Information section 4, together with the provided fitting scripts (see 10.5281/zenodo.19451754)) clearly show contributions from both molecular orientations. As these two orientations do not interact in a diluted system, it remains possible to isolate each one and fit them independently—exactly as was done in our earlier analysis to obtain the previously reported parameter sets.*

However, because the revised analysis now incorporates a full covariance treatment, both orientations are fitted simultaneously in the angular maps to provide statistically meaningful uncertainties. For clarity of presentation, the subsequent discussion of the transverse-field dependence focuses on the physical behavior itself and is not further convoluted with orientation-specific contributions, as we believe adding this extra layer of complexity would make the manuscript significantly harder to follow for a broader readership.

6. The manuscript lacks quantitative error analysis on several critical quantities: (a) Tunnel splittings Δ1,2,3(Htr): Figure 4a shows experimental data points without error bars. Given that these values are extracted from peak separations δH∥ using an approximate relation (described in the Methods section), uncertainties should be propagated and displayed. The relation provided, Δ = w1γ1+w2γ2 w1+w2 δH∥, involves weights w1,2 that are stated to be "estimated from a few frequency maps." The uncertainty in these weights directly propagates to uncertainty in the extracted Δ values.

Response: *We completely agree with the reviewer. The error bars have now been added to* ***Figure 4a,b*** *as well as to* ***SI Figure S6****. As correctly noted by the reviewer, the uncertainty in the weights* w_1*and* w_2*—or more precisely in their ratio* $k = w_2/w_1$*—propagates directly into the uncertainty of the extracted tunnel splittings* $\Delta_{1,2,3}$*. Indeed, after performing the full propagation analysis, we find that this contribution constitutes the dominant source of uncertainty in* Δ*.*

To quantify this reliably, we have re-evaluated the weights using nine independent frequency maps. The resulting uncertainties, along with the methodology, are now included in the revised ***Methods*** *section and additional Supporting Information (i.e. Origin file "Delta relation with deltaH_parallel").*

We note that using an even larger number of frequency maps would naturally reduce the associated uncertainties. However, this gradually approaches the effort required for the direct extraction of Δ *from all frequency maps (as presented in the SI video). Our aim in presenting the indirect approach was to demonstrate a rapid and minimally demanding procedure that still enables a quantitative assessment of QPI effects using only a small set of frequency maps measured at different transverse fields. This method seems to work as the error bars mentioned above lie within reasonable limits.*

7. Ligand field parameters: The quoted uncertainties (e.g., B02 = −685 } 2 MHz, representing 0.3% relative error) appear remarkably small for a fitting procedure involving multiple correlated parameters. How were these uncertainties determined? Was a proper covariance analysis performed?

Response: *We have now performed a full covariance-based error analysis using an updated and substantially refined fitting workflow (the corresponding MATLAB scripts are now provided as*

additional Supporting Information (see 10.5281/zenodo.19451754)). The ligand-field parameters obtained from both the frequency-map and angular-map fittings have been updated accordingly in the main text, and the associated fitting scripts for each dataset are included among the additional files.

As the reviewer also noted in an earlier point, two molecular orientations must be considered in the angular maps. We have now fully incorporated this into the analysis, and the updated SI includes the revised angular maps and the corresponding joint fitting routines. Importantly, the refined parameters remain very close to those reported in the previous version, so no changes were required in the physical interpretation throughout the manuscript. The key difference is that all quoted uncertainties now arise from a proper covariance analysis.

We note that, even after implementing the full covariance treatment, the resulting uncertainties remain comparable to—or in some cases smaller than—those initially reported. This stems from the fact that, despite the apparent complexity of the dataset, the individual fitting steps involve only a small number of parameters with weak mutual correlations:

1. ***Frequency-map fitting.***
   After adopting the reviewer's recommendations to remove B_{06} (given its negligible magnitude), the frequency-map fitting involves only two parameters, B_2^0 and B_4^0. The covariance matrix produced by the MATLAB fitting routine shows their correlation coefficient to be approximately −0.15, indicating only weak correlation and therefore a well-conditioned fit with largely independent parameter contributions. The dropping of B_6^0 may have caused slightly updated value of B_2^0, however no visible distinction is found in the fits compared to previous fits.
2. ***Angular-map fitting.***
   For the angular maps, all other parameters are fixed and only B_2^2 and B_4^4 are varied. The covariance matrix similarly shows weak correlation between these two parameters, again consistent with a stable and well-determined fit.

We refer the reviewer to the updated frequency- and angular-map fitting scripts, which now conform to the conventions of the most recent EasySpin version. (Older versions used a different terminology: 'CrystalOrientation' which has changed to 'SampleFrame', hence older versions are not compatible)

8. Crystal alignment: The authors state that alignment precision better than 1° is essential, but do not describe how this precision is achieved or verified experimentally.

Response: *The requirement of achieving an alignment better than 1° applies specifically to the* ***easy-axis frequency-map measurements****, where our analysis relies on the assumption that the field direction used for the "easy-axis" scans does not pick up significant contributions from transverse parameters. This precision is indeed essential, and we now explain the procedure explicitly in the revised methods section in the main manuscript (page 12, within 'step1').*

Experimentally, this alignment is achieved and verified as follows:

a) ***Geometrical pre-alignment using crystal morphology.***
   The crystal has a long, rectangular-block morphology, which allows us to align its long axis parallel to the waveguide, i.e. along the nominal $H_x$direction. This ensures that the crystallographic easy axis at least lies within the H_x–H_y plane, because the crystal must be physically rotated out of the plane (standing vertically like a pillar) for the easy axis to be out of plane—something that we do not observe most of times.
b) ***Verification using vector-field angular scans.***
   Once positioned, we record angular maps using the vector magnet. These angular maps consistently show that the easy axis is tilted by only a few degrees relative to the nominal $H_x$direction. For this reason, in the manuscript we refer to the measurement direction as

$H_{\parallel}$(the true easy-axis orientation), rather than $H_x$itself. All "easy-axis" measurements were subsequently performed along this experimentally determined $H_{\parallel}$direction, and this clarification has now been added to the updated methods section.

c) ***Experimental verification via fitting stability.***
*The fitting protocol used in the work is extremely sensitive to misalignment. If the easy axis deviates by several degrees, the fitting procedure fails in a very systematic and immediately recognizable manner. This often forces then additional tilt angle as an extra unknown variable, often leading to inconclusive fitting. We have observed such failures repeatedly during earlier, unsuccessful alignment attempts on the same molecule. As an additional remark to the referee, we would like to share that very similar attempts on another Gd-monomer based system (**Gd-Phen**) consistently failed because, in that case, the space group does not orient the easy axis parallel to any flat crystal face—making such alignment nearly impossible.*

Some of these points are now clearly explained in the revised methods section in the main manuscript (page 12, within 'step1').

9. Reproducibility: Only one crystal appears to have been measured throughout the study. Given the importance of the claims, have these observations been reproduced on independent samples? Crystal-to-crystal variations in defect concentrations, strain, or composition could affect the measured parameters.

Response: *We agree that verifying the robustness of our observations across different crystals is essential. Achieving the specific alignment conditions required for this study—namely, simultaneously obtaining near-perfect alignment of both the easy and hard (or medium) magnetic axes with two laboratory field directions—is experimentally very challenging. Nevertheless, we have succeeded in doing so very precisely on two independent crystals of the same system. The full dataset from the first sample is presented in the main article, and the corresponding measurements from the second crystal are now provided in the updated Supporting Information (see **Figure S10** in revised SI). These additional data reproduce all central features required for the analysis as compared to the frequency scan (**Figure 1d**), the angular scan, as well as the transverse-field scan (**Figure 3a,b**).*

*It is worth noting that while the easy-axis alignment is aided by the natural morphology of the crystal, the hard/medium axis does not align with any flat crystal face. For this reason, the hard-axis orientation must be maintained using plastic threads (which appear like supporting pillars at the microscopic scale), as shown in **Figure 1** of the manuscript by depicting a crystal orientation that does not lie flat on the plane (lies on the edges). This makes precise reproduction of the hard-axis angle significantly more demanding. Despite this, the features of the frequency and angular maps when only the easy-axis was aligned in-plane were highly reproducible across crystals, both in the new SI data and in earlier work by Gheorghe Taran (ref. 40), consistently yielding a unique parameter set except for the rotation-angle in the hard–medium plane.*

In addition, because the experiments are performed on 5% diluted crystals, the measured spectra arise from ensemble averaging over a very large number of Gd centers. This ensemble averaging substantially suppresses sample-to-sample variability arising from local defects, strain inhomogeneities, or microscopic disorder. As long as the dilution concentration remains unchanged, the overall spectroscopic features—including tunnel splittings, angular modulations, and field-dependent transitions and their widths—remain consistent across different crystals.

We also note that a systematic dilution-dependence study could, in principle, probe the role of strain or other broadening mechanisms. However, for the present study there is a practical limit:

- *At higher concentrations (e.g., 8–10%), the absorption features broaden significantly by dipolar intermolecular broadening, making the QPI effects difficult to resolve.*

- *At lower concentrations (<2%), the μSQUID detection sensitivity appears marginal to obtain spectra with the required signal-to-noise ratio, due to the reduced overall signal strength.*

For this reason, 5% remains the practical optimum concentration at present.

Finally, we are attempting to perform a similar study using the related ***Gd-Phen*** *molecule to test the influence of ligand environment and crystal composition. However, due to the issues mentioned while answering Q8, conducting this experiment would require substantially more time and dedicated effort.*

In summary, while the doubly constrained alignment conditions limit the number of crystals for which the full experiment can be realized, we have very precisely reproduced the key observations on a second independent sample (see ***Figure S10*** *in revised SI). We also observed that when only easy-axis is aligned, the other general spectroscopic features (the tunnel-gaps for easy-axis freq.-map, the characteristics of angular map and the obtained fitting parameters) are consistent across crystals and with earlier literature. We therefore believe the main conclusions are robust.*

10. Theoretical Framework for B44 Effects: The authors make a significant claim (lines 229–231) that their observations contradict theoretical predictions from Reference 53 (Li & Garg, 2011) regarding the motion of diabolical points with B44. Specifically, they state that Reference 53 predicted shifts of certain diabolical points toward the medium axis at large transverse fields, whereas their observations suggest shifts toward smaller transverse fields. Several concerns arise: (a) System comparison: Reference 50 treats Fe8, which has integer spin (*S* = 10) without Kramers degeneracy. The present system has a half-integer spin (*S* = 7/2) with Kramers degeneracy. The behaviour of diabolical points under transverse fields can differ qualitatively between integer and half-integer spin systems. The authors should explicitly justify why predictions from an integer-spin analysis should apply to their half-integer system.

Response: *Although Ref. 53 in the main MS (Li & Garg, 2011) specifically analyses an integer-spin system (Fe_8, S =10), the underlying theoretical framework for diabolical points (DPs) in anisotropic spin Hamiltonians is formulated for general spin S, not restricted to integer values. In combination with Phys. Rev. B 65, 064411and Gard [ref. 26 and 54] demonstrate that the overall structure of quantum-phase-interference oscillations and the evolution of DPs under transverse perturbations is qualitatively similar for both half-integer and integer spins. The total spin value is needed only to determine the multiplicity and exact positions of crossings.*

The primary qualitative difference between integer and half-integer spins concerns the Kramers degeneracy condition at zero transverse field:

- *For integer S, DPs associated with odd Δm transitions remain open (finite gap) at zero transverse field. This is the case discussed in Mn_{12} and Fe_8 studies.*
- *For half-integer S, Kramers theorem enforces that such DPs become closed (zero gap) at zero transverse field, exactly as observed experimentally in* ***Figure 4a*** *of the present manuscript.*

Apart from this distinction at $H_\perp = 0$, the response of DPs to higher-order transverse anisotropy terms—such as the B_4^4 term—is governed by the same general symmetry considerations in both integer- and half-integer-spin systems. For this reason, comparisons with the predictions of Li & Garg remain meaningful at the qualitative level. Our claim in the manuscript pertains precisely to such qualitative behavior: Ref.54 finds that increasing B_4^4 shifts certain DPs

toward the medium axis at large transverse fields, whereas our data suggest instead a shift toward smaller transverse fields, indicating a distinct trend in the present system.

Thus, while we do not expect numerical agreement between Fe_8 and our S = 7/2 system, the qualitative effect of fourth-order transverse terms on the migration of DPs is expected to be comparable, and this motivates the comparison in the discussion. We have now added explicit clarification of this point in the revised manuscript (below Eq.2, page 7, 2nd paragraph).

11. (b) Missing theoretical analysis: The authors state (line 234) that "A perturbative approach, involving spin parity and path-dependent corrections, will be pursued later." This analysis should be included, at least in the Supplementary Information, to support the claim of discovering new physics. Without theoretical justification, the disagreement with Reference 50 could equally well indicate issues with the experimental parameter extraction

Response:
The referee's concern here seems to us to be a continuation of Q. 10, and we hope that our answer there will dispel some of it. We are hesitant to discuss our calculations too much in this paper, as that would detract from the experimental focus of this paper. They are part of a larger analysis that is too technical for an experimental paper and really belong as a stand-alone theory paper. Nevertheless, a partial discussion is given in the updated section 9 of the SI and mentioned briefly at page 7 (2nd paragraph) in the main manuscript.

This added discussion indicates why the behaviour (shift of DPs with 4th order parameter) is bound to be B44-sign dependent comparing the scenarios that individually set 2nd order parameters to zero. In fact, there is no real contradiction with ref 50 (now 54), instead its complimentary as was shown by comparing the simulations for these two different cases in Fig S8(p)-(r), and (m)-(o). This aspect is now clarified further with the added discussion mentioned above. To our knowledge, the second scenario—opposite signs of B_4^4 and B_2^2—has not been explored in previous theoretical or experimental works (simply because the studied systems possessed the same signs of $B_{44}$and B_{22}). Our measurements provide the first experimental access to this regime, supported by numerical proof and the updated theoretical arguments.

12. (c) Sign dependence of B44: The authors propose a "sign-dependent role of B44" (line 231) that has not been fully explored theoretically. This is potentially interesting but requires more rigorous support than currently provided.

Response: *The 'sign-dependent role of B44' is interconnected with the mentioned 'contradiction with ref 53'. Hence, we hope the referees concern is addressed in the answers to Q10 and 11 and the added text in the updated section 9 of the SI and mentioned briefly at page 7 (2nd paragraph) in the main manuscript.*

13. Stevens operator conventions: The manuscript uses Extended Stevens operators but does not explicitly state the normalisation convention employed. Given known variations in the literature (Stevens, Wybourne, and other conventions), this should be clarified to enable reproduction of calculations.

Response: *In this work, the crystal-field Hamiltonian is expressed using the Extended Stevens Operators (ESO) as defined in [C. Rudowicz, J. Phys. C: Solid State Phys. 18, 1415 (1985)] (ref 42) and easyspin (ref. [43]), which follows the same normalization. We have now added the appropriate references.*

14. EasySpin impleentation details: The authors used EasySpin for simulations but key details are missing: Which EasySpin version was used? What specific parameters were employed for the levels function when simulating transverse field effects? The method for applying constant Htr by varying crystal orientation (θ, ϕ) as a function of H∥ (SI Section 6) is described qualitatively but no code is provided. Simulation scripts should be made available.

Response: *All simulation, fitting scripts are now provided. These include: (1) simulation of Zeeman diagrams with or without transverse fields, (2) Fitting of frequency map, (2) Fitting of angular map, (3) Simulation of transverse field map, (4) Simulation of Delta vs transverse field, (5) Script for DP search (surface plots) see [10.5281/zenodo.19451754]. These scripts are compatible with most updated EasySpin version (we used Easyspin 6.0.6). Please note that older EasySpin versions followed a different terminology ('CrystalOrientation' which has changed to 'SampleFrame'), hence is not compatible.*

15. Comparison with Reference 40: The relationship between the present study and the prior µSQUID-EPR work on the same compound (Taran et al., 2023) should be clarified. Are the ligand field parameters consistent between the two works? What new physics does the present study reveal beyond Reference 40?

Response: *Now clarified in page 3 (in the second paragraph following equation 1).*

16. Equation (2) validity: Equation (2) for the oscillation period is stated to apply "with only one axial and one transverse parameter in the Hamiltonian." The actual Hamiltonian includes B04 and B44 terms. How valid is this approximation, and what corrections should be expected?

Response: *We apologise for leading to this confusion. We tried to imply that it is an incomplete description if we use only one axial and one transverse term, however it yields a basic estimation of the order of magnitude (given that the contribution of the B_2^2 term is eventually found much more significant than that of B_4^4 in this system). This is now mentioned in the updated main article. Please note that later in the following paragraph we mention again that this theoretical estimation is not sufficient, and we need a more complete theory, that would be the 'path dependent perturbation-based theory for this system (to follow as a separate publication).*

One can also see the simulations with and without B_4^4 terms to comprehend that this expression is not sufficient but can provide an estimate of order of magnitude of the oscillation period (see ***Figure S8****, particularly panels c-i).*

17. Parameter B06: The value B06 = 0.004 □} 0.004 MHz quoted in the SI has 100% relative uncertainty, making its physical significance questionable. Should this term be included in the analysis at all?

Response: *We agree with the reviewer that this term can be dropped allowing simplicity of analysis, as updated in the main article.*

18. Please provide a detailed explanation for the four-order-of-magnitude discrepancy between CASSCF-predicted (B22 = 0.037 MHz) and experimentally determined (B22 = −275 MHz) values for the transverse second-order parameter.

Response: *Please see responses to question 1.*

19. What physical mechanism generates the large rhombic anisotropy (B22/B02 ≈ 0.4) in an L = 0 ion? What structural distortions from D4h symmetry are responsible?

Response: *Please see responses to question 1.*

20. How are contributions from the two crystallographically distinct molecular orientations (related by +x,−y,−z symmetry) separated in the analysis? Could interference between signals from the two orientations contribute to the observed features?

Response: *Please see responses to question 1 to 5*

21. Please provide error bars for Δi(Htr) data in Figure 4 and explain how uncertainties were estimated, including propagation of errors from the weights w1,2.

Response: *This is now included in the updated manuscript. Please see response to question 6.*

22. Can you provide the perturbative analysis for the sign-dependent B44 effects, or explain why the predictions of Reference 50 (derived for integer spin) should or should not apply to your half-integer spin system?

Response: *Please see response to question 10-12.*

23. Have measurements been repeated on additional crystals to confirm reproducibility?

Response: *Yes, see response to question 9.*

24. What is the estimated spin temperature during microwave irradiation, and how might thermal population effects influence the observed tunnel splittings and their transversefield dependence?

Response: *As updated in the main article (page 3 paragraph 1), there is significant role of thermal population as spin temperature is raised to several hundreds of milliKelvin (very likely about ~ 700 ±100 mK as we are developing the route of estimation). Presently we lack a precise estimation of spin temperature, since unlike regular EPR, where the expression of 'absorbance' is well known in terms of bath temperature, the 'magnetization peaks' do not yet have an analytical (or Hamiltonian matrix-elements based) expression and is under development.*

However the tunnel splittings are more fundamental, they depend on the QPI effects by transverse and axial LF parameters. We expect negligible change in these parameters for such an order of spin temperature raise. The crystal or molecular structure supposedly do-not undergo large deformations by changing only a few hundreds of mK; in contrast, cooling the system from room temperature to low temperature can introduce gradual (or even abrupt) structural changes,

and at sub-Kelvin temperatures the system is likely frozen into a configuration that departs from its room-temperature structure (XRD), as discussed earlier.

Technical Corrections

1. Line 4: Author affiliation superscripts should be verified for consistency

Response: *Done*

2. Line 115: “8.90 GHz” should be consistent with “8.9 GHz” used elsewhere.

Response: *This is now updated in Fig 1 and 8.90 is consistently used throughout.*

3. Figure 1 caption: The transition labelling (Δ_1: ms = −5/2 → +1/2) should clarify whether *these are diabatic or adiabatic state labels.*

Response: *Here m_s are the diabatic state labels, while the actual Hamiltonian (with all transverse terms) mixes those basis states. Therefore, the true eigenstates are adiabatic states (i.e. eigenstates of the full Hamiltonian), and their dominant component may approximately correspond to an m_s value, but not strictly. This is now clarified in main article page 4*

4. SI Page 4: The fit uncertainty for B0 6 = 0.004□}0.004 MHz (100% relative error) questions the significance of including this term.

Response: *This parameter has been now excluded.*

On behalf of all co-authors, I would like to thank the referees for taking the time to carefully review our manuscript and for their valuable comments and suggestions, which undoubtedly will contribute to the betterment of the manuscript. Bellow we comment on the minor points raised by referee 3. We hope the MS is now suitable for publication.

Response to the Referee 3

We thank the reviewer for the positive reply and acceptance. The comments raised appear to be indications of editorial reverification of the updated information, mainly concerning supplementary information. Hence in our replies below, we intend to support the editor with a clarified explanation of the updated changes in our last round.

Reviewer #3 (Remarks to the Author):

Dear Editor,

I have carefully reviewed the revised manuscript and the authors' point-by-point response to my original comments. Overall, the revision represents a substantial improvement, and the authors have addressed most of my concerns responsibly and in good faith.

Two residual concerns remain, which I flag for the editor's consideration rather than as blocking objections. First, regarding the comparison with Li et Garg (ref. 53) and the proposed sign-dependent role of B44: the authors acknowledge that a full perturbative treatment is deferred to a forthcoming theoretical publication. The discussion added to Section 9 of the SI and the numerical evidence from Figure S8 provide a reasonable basis for the claims made, and the authors are careful to frame the theoretical comparison as qualitative. The manuscript language is now sufficiently hedged that I consider this acceptable for an experimental paper, provided the SI discussion is indeed as detailed as described in the rebuttal — I was not able to verify the revised SI directly, and I would ask the editor to confirm this.

Response: *To further clarify and guide the editor, we refer to the additional discussion provided in Section 9 of the Supporting Information (SI). In particular, Figure S9 and the accompanying discussion at the end of this section extend the qualitative reasoning by clearly distinguishing between two limiting scenarios. This framework establishes 'Case IIa' and 'Case IIb' (in Figure S9) as the only two extreme possibilities. Consequently, it provides a consistent and logical basis for understanding how the introduction of a sign-dependent* $B44$ *term (the fourth-order transverse anisotropy) drives the system to evolve continuously from 'Case I' toward either 'Case IIa' or 'Case IIb', depending on the relative signs involved.*

Second, regarding Figure S10: the rebuttal states that this figure reproduces the key features required for the analysis, including the transverse-field oscillations. If the oscillations in Delta(Htr) are clearly visible in the second-crystal data, reproducibility of the central observation is established; if only the easy-axis frequency map is shown, this point remains partially open.

Subject to editorial confirmation that the revised SI contains the transverse-field oscillation data for the second crystal and an adequately detailed Section 9, I recommend acceptance of the revised manuscript. The μSQUID-EPR methodology and the direct observation of Berry phase interference in a 4f molecular magnet represent a genuine experimental advance, and the paper is appropriate for publication in this journal.

Response: *To further clarify and guide the editor, we refer to Figure S10 in the Supporting Information. This figure demonstrates reproducibility in a second sample not only through the easy-axis frequency map (Figure S10a), but also by capturing the transverse-field-dependent oscillatory features (Figure S10b,c) of tunnel-splittings under identical experimental conditions. The oscillations are clearly resolvable and exhibit the same characteristic pattern as those shown in Figure 3 of the main text, confirming reproducibility of the central observation regarding topological suppression.*

Referee Report for Nature Communications

Manuscript Title: Topological Suppression of Quantum Tunnelling in a Lanthanide Single-Ion Molecular Magnet

This manuscript reports the direct spectroscopic observation of quantum phase interference (QPI) and parity-dependent oscillations in tunnel splittings in a gadolinium-based single-ion molecular magnet, $[^{160}GdPc_2]^-$. The authors employ a combined μSQUID-EPR technique to map the complete spin manifold ($S = 7/2$) under transverse magnetic fields, resolving avoided level crossings that reveal topologically quenched quantum tunnelling of magnetisation (QTM). The work claims to demonstrate the first direct observation of Berry phase effects in a 4f-based molecular magnet and highlights the significant role of fourth-order transverse ligand field parameters (B_4^4) in determining the positions of diabolical points.

The manuscript addresses an important gap in the field of molecular magnetism. While QPI has been observed in 3d-based molecular magnets (Fe_8, Mn_{12}) through Landau-Zener tunnelling experiments, extending these observations to lanthanide systems has remained challenging due to the larger tunnel gaps that preclude conventional approaches. The μSQUID-EPR methodology presented here represents a genuine technical advance that enables direct spectroscopic access to tunnel splittings in 4f systems.

If the claims are validated, this work would constitute a significant contribution with implications for understanding spin topology in lanthanide-based quantum systems, rational design of molecular qubits with controlled tunnelling rates, and development of holonomic quantum computation schemes using molecular magnets.

However, several critical issues must be addressed before publication can be recommended.

Major Concerns

1. Significant Discrepancy Between CASSCF Calculations and Experimental Parameters

This is the most significant concern with the manuscript. Table S2 in the Supplementary Information reports crystal field parameters extracted from CASSCF-SO calculations, while the main text provides experimentally determined values. A careful comparison reveals substantial inconsistencies.

For the axial parameters, the agreement is reasonable: the CASSCF value of $B_2^0 = -811$ MHz compares to the experimental value of -685 MHz (ratio ~ 1.2), and $B_4^0 = -0.239$ MHz from CASSCF compares to -1.45 MHz experimentally (ratio ~ 6).

However, for the transverse parameters, the discrepancies are far more severe. The CASSCF calculation yields $B_2^2 = 3.73\times10^{-2}$ MHz, whereas the experimental fitting requires $B_2^2 = -275$ MHz. This represents a difference of approximately **four orders of magnitude** ($\sim 7400\times$). Similarly,

for B_4^4, the CASSCF value of 0.160 MHz differs from the experimental value of 3.9 MHz by a factor of ~ 24.

This massive discrepancy for the transverse second-order parameter B_2^2 is not discussed anywhere in the manuscript and represents a fundamental problem. The transverse parameters are precisely those responsible for the QPI effects that are the central focus of this work. The authors cannot simultaneously claim that CASSCF calculations support their model while the most critical parameters for QPI observation differ by nearly four orders of magnitude.

The authors must provide a detailed explanation for this discrepancy. Possible explanations might include: (a) limitations of the CASSCF method in capturing higher-order spin-orbit effects that generate transverse anisotropy in $L = 0$ ions; (b) environmental effects (crystal packing, counter-ion interactions) not captured by the single-molecule calculation; or (c) issues with the experimental parameter extraction procedure. Whatever the explanation, it must be explicitly discussed and justified.

2. Physical Justification for Large Transverse Anisotropy in Gd^{3+}

Gadolinium(III) possesses a $^8S_{7/2}$ ground state with $L = 0$, meaning that first-order spin-orbit coupling cannot generate magnetic anisotropy. Any zero-field splitting must arise from higher-order effects, specifically through mixing with excited ^6P states via spin-orbit coupling combined with the crystal field. This is well-established physics.

However, the reported experimental value of $B_2^2 = -275$ MHz represents approximately 40% of the axial parameter $B_2^0 = -685$ MHz. Such a large rhombic-to-axial ratio is unusual and requires physical justification. The local coordination environment around Gd^{3+} in the double-decker phthalocyanine complex is approximately D_{4h} (or lower, C_4). In strict D_{4h} symmetry, the B_2^2 term should vanish by symmetry. While slight distortions can lift this restriction, a value of B_2^2 that is 40% of B_2^0 implies substantial symmetry lowering.

Questions for the authors:

- What structural distortions from ideal D_{4h} symmetry are responsible for the large B_2^2 term?
- Reference 40 (Taran et al., 2023) is cited for prior determination of these parameters. Did that work provide physical justification for these values?
- Can molecular dynamics or DFT calculations provide insight into the local symmetry breaking?

3. Treatment of Two Crystallographically Distinct Molecular Orientations

Figure S1 clearly shows that two differently oriented $[^{160}GdPc_2]^-$ molecules exist within the unit cell, related by a $(+x, -y, -z)$ symmetry operation. The authors explicitly note that "the hard and medium axes are nearly 90° apart from each molecule."

This is a critical issue that is not adequately addressed in the manuscript. When the magnetic field is applied along what is the "hard axis" for one molecular orientation, it will be close to the "medium axis" for the other orientation. Since QPI effects are strongly dependent on the

direction of the transverse field relative to the molecular axes, the two orientations should exhibit qualitatively different behaviour.

Specific concerns:

- How do the authors distinguish or separate contributions from the two molecular orientations in their μSQUID-EPR data?
- When mapping $\Delta_i(H_{\mathrm{tr}})$ oscillations, do both orientations contribute simultaneously? If so, how is this deconvoluted?
- Could the observed "tiling pattern" near $H_{||} = 0$ (Figures 3a,b) arise partially from superposition of signals from the two orientations rather than purely from QPI effects?
- The fitting of angular maps (Figure S4) should show evidence of two sets of transitions if both orientations contribute. Is this observed?

4. Error Analysis and Reproducibility

The manuscript lacks quantitative error analysis on several critical quantities:

(a) Tunnel splittings $\Delta_{1,2,3}(H_{\mathrm{tr}})$: Figure 4a shows experimental data points without error bars. Given that these values are extracted from peak separations $\delta H_{||}$ using an approximate relation (described in the Methods section), uncertainties should be propagated and displayed. The relation provided, $\Delta = \frac{w_1\gamma_1+w_2\gamma_2}{w_1+w_2}\delta H_{||}$, involves weights $w_{1,2}$ that are stated to be "estimated from a few frequency maps." The uncertainty in these weights directly propagates to uncertainty in the extracted Δ values.

(b) Ligand field parameters: The quoted uncertainties (e.g., $B_2^0 = -685 \pm 2$ MHz, representing 0.3% relative error) appear remarkably small for a fitting procedure involving multiple correlated parameters. How were these uncertainties determined? Was a proper covariance analysis performed?

(c) Crystal alignment: The authors state that alignment precision better than 1° is essential, but do not describe how this precision is achieved or verified experimentally.

(d) Reproducibility: Only one crystal appears to have been measured throughout the study. Given the importance of the claims, have these observations been reproduced on independent samples? Crystal-to-crystal variations in defect concentrations, strain, or composition could affect the measured parameters.

5. Theoretical Framework for B_4^4 Effects

The authors make a significant claim (lines 229–231) that their observations contradict theoretical predictions from Reference 50 (Li & Garg, 2011) regarding the motion of diabolical points with B_4^4. Specifically, they state that Reference 50 predicted shifts of certain diabolical points toward the medium axis at large transverse fields, whereas their observations suggest shifts toward smaller transverse fields.

Several concerns arise:

(a) System comparison: Reference 50 treats Fe_8, which has integer spin ($S = 10$) without Kramers degeneracy. The present system has half-integer spin ($S = 7/2$) with Kramers degeneracy. The behaviour of diabolical points under transverse fields can differ qualitatively between integer and half-integer spin systems. The authors should explicitly justify why predictions from an integer-spin analysis should apply to their half-integer system.

(b) Missing theoretical analysis: The authors state (line 234) that "A perturbative approach, involving spin parity and path-dependent corrections, will be pursued later." This analysis should be included, at least in the Supplementary Information, to support the claim of discovering new physics. Without theoretical justification, the disagreement with Reference 50 could equally well indicate issues with the experimental parameter extraction.

(c) Sign dependence of B_4^4: The authors propose a "sign-dependent role of B_4^4" (line 231) that has not been fully explored theoretically. This is potentially interesting but requires more rigorous support than currently provided.

Minor Concerns

6. Stevens operator conventions: The manuscript uses Extended Stevens operators but does not explicitly state the normalisation convention employed. Given known variations in the literature (Stevens, Wybourne, and other conventions), this should be clarified to enable reproduction of calculations.

7. EasySpin implementation details: The authors used EasySpin for simulations but key details are missing: Which EasySpin version was used? What specific parameters were employed for the `levels` function when simulating transverse field effects? The method for applying constant H_{tr} by varying crystal orientation (θ, ϕ) as a function of $H_{||}$ (SI Section 6) is described qualitatively but no code is provided. Simulation scripts should be made available.

8. Comparison with Reference 40: The relationship between the present study and the prior μSQUID-EPR work on the same compound (Taran et al., 2023) should be clarified. Are the ligand field parameters consistent between the two works? What new physics does the present study reveal beyond Reference 40?

9. Equation (2) validity: Equation (2) for the oscillation period is stated to apply "with only one axial and one transverse parameter in the Hamiltonian." The actual Hamiltonian includes B_4^0 and B_4^4 terms. How valid is this approximation, and what corrections should be expected?

10. Parameter B_6^0: The value $B_6^0 = 0.004 \pm 0.004$ MHz quoted in the SI has 100% relative uncertainty, making its physical significance questionable. Should this term be included in the analysis at all?

Specific Questions for Authors

1. Please provide a detailed explanation for the four-order-of-magnitude discrepancy between CASSCF-predicted ($B_2^2 = 0.037$ MHz) and experimentally determined ($B_2^2 = -275$ MHz) values for the transverse second-order parameter.

2. What physical mechanism generates the large rhombic anisotropy ($B_2^2/B_2^0 \approx 0.4$) in an $L = 0$ ion? What structural distortions from D_{4h} symmetry are responsible?
3. How are contributions from the two crystallographically distinct molecular orientations (related by $+x, -y, -z$ symmetry) separated in the analysis? Could interference between signals from the two orientations contribute to the observed features?
4. Please provide error bars for $\Delta_i(H_{\text{tr}})$ data in Figure 4 and explain how uncertainties were estimated, including propagation of errors from the weights $w_{1,2}$.
5. Can you provide the perturbative analysis for the sign-dependent B_4^4 effects, or explain why the predictions of Reference 50 (derived for integer spin) should or should not apply to your half-integer spin system?
6. Have measurements been repeated on additional crystals to confirm reproducibility?
7. What is the estimated spin temperature during microwave irradiation, and how might thermal population effects influence the observed tunnel splittings and their transverse-field dependence?

Technical Corrections

- Line 4: Author affiliation superscripts should be verified for consistency.
- Line 115: "8.90 GHz" should be consistent with "8.9 GHz" used elsewhere.
- Figure 1 caption: The transition labelling (Δ_1: $m_s = -5/2 \to +1/2$) should clarify whether these are diabatic or adiabatic state labels.
- SI Page 4: The fit uncertainty for $B_6^0 = 0.004 \pm 0.004$ MHz (100% relative error) questions the significance of including this term.
